# Safety and effectiveness of SOFIA/SOFIA PLUS for direct aspiration as first line treatment in patients with acute anterior ischemic stroke: results from the prospective, multicentric SESAME study

**DOI:** 10.3389/fneur.2024.1441810

**Published:** 2024-09-25

**Authors:** Ulf Neuberger, Gaultier Marnat, Xavier Barreau, Antonio Pitrone, Antonio A. Caragliano, Monika Killer-Oberpfalzer, Johannes A. R. Pfaff, Christoph J. Maurer, Ansgar Berlis, Reinoud Bokkers, Maarten Uyttenboogaart, Nader Sourour, Frédéric Clarençon, Fritz Wodarg, Christophe Cognard, Georg Bohner, Johannes Trenkler, Laurent Spelle, Werner Weber, Nasreddine Nouri, Susanne Bonekamp, Götz Thomalla, Jens Fiehler, Martin Bendszus, Markus A. Möhlenbruch

**Affiliations:** ^1^Department of Neuroradiology, Heidelberg University Hospital, Heidelberg, Germany; ^2^Department of Neuroradiology, Pellegrin Hospital, Bordeaux, France; ^3^Department of Neuroradiology, Policlinico Martino University Hospital, Messina, Italy; ^4^Department of Neurology/Institute of Neurointervention, Christian Doppler Medical Center, Paracelsus Medical University, Salzburg, Austria; ^5^Department of Neuroradiology, University Hospital Salzburg, Paracelsus Medical University, Salzburg, Austria; ^6^Department of Diagnostic and Interventional Neuroradiology, University Hospital Augsburg, Augsburg, Germany; ^7^Department of Neuroradiology, University of Groningen Hospital, Groningen, Netherlands; ^8^Department of Neuroradiology, Pitié Salpêtrière University Hospital, Paris, France; ^9^Department of Neuroradiology, University Hospital Kiel, Kiel, Germany; ^10^Department of Neuroradiology, Purpan Hospital, Toulouse, France; ^11^Department of Neuroradiology, Charité Berlin Hospital, Berlin, Germany; ^12^Department of Neuroradiology, Kepler University Hospital, Linz, Austria; ^13^Department of Neuroradiology, Bicêtre Hospital, Paris, France; ^14^Department of Diagnostic and Interventional Radiology, Neuroradiology and Nuclear Medicine, Bochum University Hospital, Bochum, Germany; ^15^Department of Neuroradiology, Salengro Hospital, Lille, France; ^16^Department of Neurology, University Medical Center Hamburg Eppendorf, Hamburg, Germany; ^17^Department of Neuroradiology, University Medical Center Hamburg Eppendorf, Hamburg, Germany

**Keywords:** stroke, thrombectomy, aspiration (MeSH), good clinical practice (GCP), prospective observational study

## Abstract

**Background:**

Mechanical thrombectomy is a cornerstone treatment for acute ischemic stroke (AIS) with large vessel occlusion (LVO), yet the optimal technique remains debated. The SOFIA/SOFIA PLUS catheter has emerged as a promising tool for direct aspiration thrombectomy.

**Purpose:**

This prospective multi-center study, adhering Good-Clinical-Practice guidelines, aimed to evaluate the safety and efficacy of the SOFIA/SOFIA PLUS catheter for direct aspiration as a first-line treatment technique in patients with acute anterior circulation LVO.

**Materials and methods:**

Between 10/2017 and 12/2021, 246 consecutive patients presenting with AIS due to anterior circulation LVO were enrolled from 14 European centers. Primary treatment with SOFIA catheters was performed within 6 h of symptom onset. Clinical and radiological data were collected, and statistical analyses were conducted.

**Results:**

The mean age of the included patients was 71.6 ± 13.9 years, with 44.7% being male. Primary aspiration achieved complete recanalization in 72.8% of patients, with functional independence observed in 63.8% after 90 days. Secondary outcomes included a median NIHSS of 4 at 24 h post-procedure, median ASPECTS of 7 on follow-up imaging, and a mortality rate of 24.4% at 90 days. No device malfunctions were observed, and the rate of symptomatic intracranial hemorrhage was 4.4%.

**Conclusion:**

Primary aspiration with the SOFIA/SOFIA PLUS catheter demonstrates favorable safety and efficacy profiles in the treatment of anterior circulation LVO. These findings support the utilization of this technique as a first-line approach in mechanical thrombectomy for AIS, contributing to the growing body of evidence endorsing the effectiveness of direct aspiration thrombectomy in stroke management.

## Introduction

Acute ischemic stroke (AIS) is the leading cause of long-term disability in Europe, affecting over a million individuals each year (1). The economic burden of stroke is substantial, and likewise, long-term impairment associated with stroke is significant, as more than 50% of stroke patients require discharge to rehabilitation or skilled nursing care. Notably, emergent large vessel occlusion (LVO), mainly in the internal carotid artery (ICA) or middle cerebral artery accounts for over 30% of all AIS cases (2).

In addition to the intravenous administration of tissue plasminogen activator for the treatment for AIS, recent trials demonstrated a significantly improved clinical outcome after mechanical thrombectomy in patients with large vessel occlusion (3).

Prompt recanalization of the occluded artery represents a crucial factor in enhancing clinical outcomes for patients with AIS, as rapid reperfusion plays a vital role in preventing damage to the penumbral region, resulting in improved neurological outcomes with fewer deficits and a notable reduction in stroke-related mortality and morbidity (4–6).

Recently, the direct-aspiration first-pass technique, performed with advanced aspiration catheters, has shown promising results in both retrospective and prospective studies (7, 8). These distal aspiration systems offer excellent navigability and have achieved high recanalization rates with low morbidity and favorable functional outcomes (9–11). Even though the direct contact aspiration technique achieved high final recanalization rates, the use of adjunctive devices or rescue procedures in these studies was frequent, limiting evaluation of this technique alone (12–15).

Here, we investigated the safety and efficacy of a first-line strategy using a distal aspiration system for thrombectomy as first-line approach. The primary objective of this prospective, multi-center study was to assess the safety and efficacy of the SOFIA and SOFIAPLUS catheter for direct aspiration as a first line treatment technique (SESAME) in patients with acute ischemic stroke from LVO. This was the first prospective Good Clinical Practice study applying this device.

## Methods

Safety and efficacy of the SOFIA/SOFIA PLUS catheter for direct aspiration as a first-line treatment technique was a European multi-center, prospective, single-arm, observational registry study (https://classic.clinicaltrials.gov/ct2/show/NCT03417349, ClinicalTrials.gov Identifier: NCT03417349). The study protocol of SESAME can be found in the Supplementary material. It aimed to evaluate the quickness, efficacy and safety of the use of SOFIA and SOFIA PLUS catheters for contact aspiration as a first-line treatment in patients with acute ischemic stroke of the anterior circulation due a large vessel occlusion (LVO). Each patient was screened for eligibility and documented in a site-specific screening protocol.

All procedures were in accordance with national and local ethical and institutional guidelines and routine clinical practice at each site. Local Ethics Committees approved the study, and the patients or their representatives provided written informed consent, according to local regulations. Safety endpoints were adjudicated for severity and causality by an independent Clinical Events Committee (CEC). All data were monitored through on-site visits.

### Study device

The Soft Torqueable Catheter Optimized For Intracranial Access (SOFIA) and SOFIA PLUS catheters are single lumen, flexible catheters, designed with coil and braid reinforcement. Specifically, the SOFIA catheter has an outer diameter of 5F and an inner diameter of 0.055 in, and the SOFIA PLUS has an outer diameter of 6F and an inner diameter of 0.070 in. Both catheters have a soft distal tip, and the tip is steam-shapeable and the proximal shaft is torqueable for distal navigation. The coil and braid construction provide kink resistance and 1:1 push/pull control. Once navigated to the site of the occlusion, the SOFIA and SOFIA PLUS catheters can be used in conjunction with an aspiration source, such as a pump or syringe, to facilitate aspiration thrombectomy of the occluded vessel.

### Study population

All consecutive patients presenting with acute ischemic stroke between October 1st, 2017 and December 31th, 2021 were assessed prior to study enrolment, based on the patient’s medical condition and available diagnostic screening procedures. Eligibility criteria included age of at least 18 years, presentation with a large vessel occlusion of the anterior circulation (within the internal carotid artery and internal carotid terminus, middle cerebral artery M1/M2 and anterior cerebral artery A1/A2 segments) resulting in a National Institute of Health Stroke Scale (NIHSS) of at least 2 but not exceeding 29, and onset or last known to be well within 6 h. Patients were also required for inclusion to have a pre-morbid Rankin Scale of 1 or less. Exclusion criteria included the presence of intracranial hemorrhage and evidence of a large infarct core, defined by extensive early ischemic changes in the Alberta Stroke Program Early CT score (ASPECTS) of less than 6.

### Clinical evaluation

Baseline epidemiological and clinical characteristics were collected by independent stroke neurologists included sex, age, pre-morbid modified Rankin Scale (mRS) score and history of risk factors, such as hypertension, hyperlipidemia, diabetes mellitus, ischemic heart disease, atrial fibrillation, or previous stroke. Physical examinations, including admission blood pressure and heart rate, as well as routine practice lab results were recorded. Severity of stroke symptoms measured by NIHSS as well as the patient’s functional status, prior and after stroke onset, measured by mRS scores were evaluated during independent neurological assessments by qualified personnel. All concomitant medications potentially affecting stroke were captured (ASA, anticoagulants, antiplatelets, and antihypertensive medications).

### Imaging analysis and treatment

Baseline imaging was performed using either non-contrast enhanced CT and contrast-enhanced CT angiography or MRI (± contrast-enhanced MR angiography) dependent on the institutional standard. Thrombus length was estimated on baseline imaging by the core lab. If applicable, intravenous thrombolysis was applied, according to national and international guidelines.

Angiographic treatment consisted of placement of a guide catheter or a balloon guide catheter in the supplying internal carotid artery according to the institutional standard. Subsequently, a SOFIA or SOFIA PLUS catheter for direct aspiration thrombectomy was advanced to the occlusion site. The choice to use either catheter was left at the discretion of the operator. Once navigated to the site of the occlusion, the SOFIA or SOFIA PLUS catheters were used in conjunction with an aspiration source, either a pump or a syringe, to perform aspiration thrombectomy of the occluded vessel. After a maximum of three unsuccessful aspiration attempts, interventionalists were advised to switch to a different technique. However, the operator was authorized to switch to another technique after the first pass if deemed necessary. The core lab had access to all angiographic data to assess procedural results and complications.

### Overview of primary and secondary endpoints

The primary endpoint of the study was the proportion of patients with a good clinical outcome, defined as mRS ≤ 2 at 90 days post-treatment, as assessed by an independent certified neurologist.

Secondary endpoints included the change of the NIHSS score at 24 h and at discharge, and quality of life (PROMIS Scale v1.2 questionnaire) at 90 days. Safety endpoints included any new stroke, intracranial hemorrhage (ICH) including symptomatic ICH (sICH) according to the Heidelberg Bleeding Classification (16), device-and procedure-related adverse events (such as vessel perforation, vessel dissection, or embolization of thrombus in a previously unaffected vascular territory, i.e., thrombus in the territory of the anterior cerebral artery after initial occlusion of the middle cerebral artery territory) within 90 days of follow up. Imaging endpoints were assessed by a central core lab blinded to all data (EppData, Hamburg) and included assessment of the modified Thrombolysis in Cerebral Infarction (mTICI) score after first line treatment and at the end of the procedure. Furthermore, the first-pass effect (FPE) was assessed, defined as attaining mTICI 2c or 3 after the first aspiration maneuver. Adjudication of symptomatic intracranial hemorrhages in follow-up imaging 24 h (± 12 h) after the procedure using native cranial CT was performed by a clinical event committee after consideration of all clinical data.

### Statistical analysis

Descriptive statistical analyses were summarized for the demographic, clinical and procedural parameters at baseline, using conventional metrics such as number of observations, mean, median, standard deviation, and interquartile range for continuous variables and counts as well as percentages for discrete variables. Metrics were tested for normality using the Shapiro–Wilk test, and Wilcoxon rank-sum test or *t*-test were then used to assess differences between the metrics of patients that were either treated primarily with the SOFIA or SOFIA PLUS catheter. Confidence intervals were presented two-sided. Statistical tests were performed with a two-tailed design and a significance level of 0.05. The clinical endpoint of the study (mRS after 90 days) was not available for all patients (*n* = 34/246), and could not be retrieved at the time of the analysis. Since the simple exclusion of cases with missing values can potentially lead to severely misleading results (17), we have used multiple imputation as the method of choice to supplement missing clinical data. Data imputation was performed with the “missForest” package of R, which is especially used to impute missing values of mixed-type (continuous and/or categorical) and non-normally distributed data. It uses a random forest model that is trained on the observed values of a data matrix to predict the missing values.

Analyses were performed using R version 4.1.3 (Foundation for Statistical Computing, Vienna, Austria).

## Results

**Figure 1:**
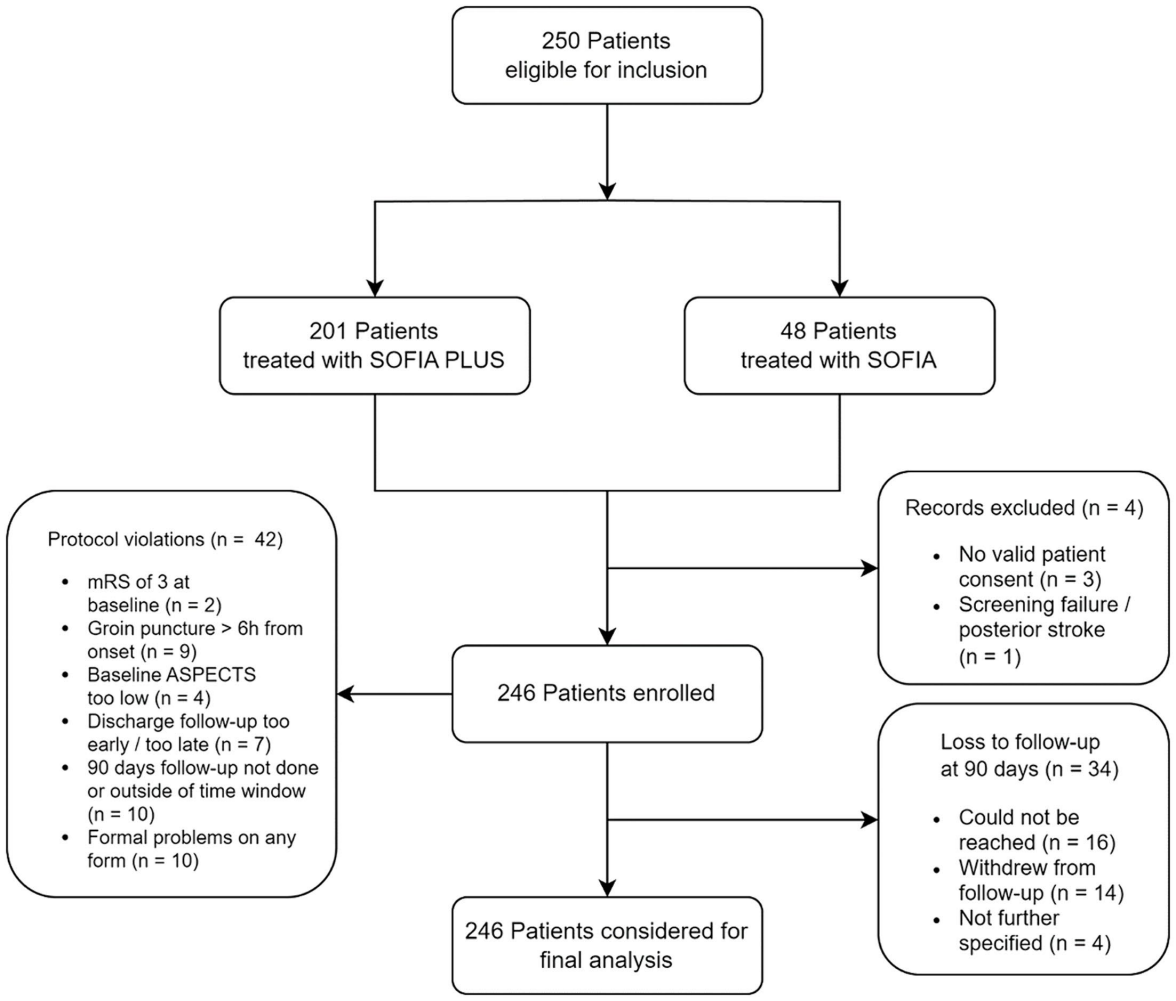
Flowchart of patients in the SESAME trial. mRS, modified Rankin Scale; ASPECTS, Alberta Stroke Program Early CT Score. Choice to use either SOFIA or SOFIA PLUS was at left at the discretion of the operator.

Overall, 250 patients were enrolled in the SESAME trial between October 1st, 2017 and December 31st, 2021 across 14 European sites (five from Germany, five from France, two from Austria, one from Italy, and one from Netherlands). Since core laboratory endpoint data were not available for four patients, they were required to be omitted from the analysis, resulting in a final analysis cohort of 246 patients (see Figure 1 for a flowchart of patient inclusion).

Baseline demographic, clinical and radiological data are summarized in Table 1. The mean age of patients was 71.6 years (± 13.9) with 44.7% being male. On admission, patients presented with a median NIHSS of 14 (IQR 10–18), and a median ASPECTS of 8 (IQR 7–10). Intravenous thrombolysis was administered in 117 patients (47.5%). Occlusions were located at the distal ICA and/or carotid T (15.3%), the M1 segment (75.6%) or M2 segments (8.9%). Mean onset-to-groin time was 252.0 min ± 172.4 min.

**Table 1:** Baseline descriptive statistics for all enrolled patients, along with comparative statistics for patient cohorts using SOFIA and SOFIA 6F PLUS.

	Complete cohort	6F (*n* = 198)	5F (*n* = 48)	*p* value
*N*	246	198 (80.5)	48 (19.5)	n. a.
Age	71.6 + − 13.9	71.5 + − 13.7	71.7 + − 14.4	0.949
Sex (male)	110 (44.7)	93 (47.0)	17 (35.4)	0.152
Right-sided stroke	131 (53.2)	105 (53.0)	26 (54.2)	0.996
IV Lysis	117 (47.5)	90 (45.5)	27 (56.3)	0.181
Transfer from external hospital	83 (33.7)	68 (34.3)	15 (31.3)	0.716
Admission NIHSS	14 (10–18)	14 (10–18)	14 (9–17)	0.149
Distal ICA or carotid T occlusions	36 (14.6)	34 (17.2)	2 (4.2)	0.077
M1 occlusion	186 (75.6)	150 (75.8)	36 (75.0)	0.911
M2 occlusion	22 (8.9)	13 (6.6)	9 (18.8)	0.017
Baseline ASPECTS	8 (7–10)	8 (7–10)	8 (7–10)	0.357
Thrombus length (mm)	8.0 + − 6.0	8.7 + − 6.0	5.1 + − 4.7	0.002
Time from onset to groin puncture	252.0 + − 172.4	257.0 + − 183.3	230.1 + − 115.2	0.788

Metrics are listed as median (IQR) or mean ± standard deviation for continuous variables, and number (%) for ordinal variables. NIHSS, National Institute of Health Stroke Scale; ICA, Internal carotid artery; and ASPECTS, Alberta Stroke Program Early Computed Tomography Scores.

The majority of patients in the study (80.5%, *n* = 198) were initially treated with the SOFIA PLUS 6F catheter, whereas in a smaller proportion (19.5%, *n* = 48) the SOFIA catheter was used as first device. In three cases, the SOFIA PLUS catheter was initially used but later replaced by the SOFIA catheter, as a sufficient position for contact aspiration could not be established with the larger catheter. Overall, 82 individuals (33.3%) underwent treatment under general anesthesia, while the remainder were treated under local anesthesia solely or conscious sedation. For second-line treatment after a mean of *n* = 2.00 ± 1.35 aspiration maneuvers, stent retrievers were used in 19.1% (*n* = 47) of patients. Among the 199 patients who were treated with primary aspiration alone, the distribution of passes was as follows: 122 patients (61.3%) had a single pass, 68 patients (34.2%) required two passes, and 17 patients (8.5%) needed three passes. Additionally, five patients (2.5%) underwent more than three passes.

For the 47 patients who received a second-line technique, the distribution was: nine patients (19.1%) had a single pass, 18 patients (38.3%) required two passes, and nine patients (19.1%) needed three passes, while 11 patients (23.4%) underwent more than three passes.

### Primary outcome

**Figure 2:**
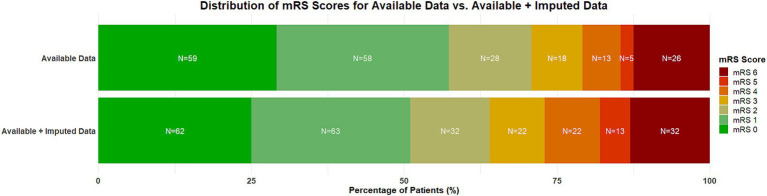
Distribution of mRS scores at 3 months of available data (*n* = 212) and the dataset after imputation (*n* = 246). A score of 0 on the mRS indicates no symptoms, a score of 1 indicates no clinically significant disability, a score of 2 indicates slight disability (patients are able to look after their own affairs without assistance but are unable to carry out all previous activities), a score of 3 indicates moderate disability (patients require some help but are able to walk unassisted), a score of 4 indicates moderately severe disability (patients are unable to attend to bodily needs without assistance and are unable to walk unassisted), a score of 5 indicates severe disability (patients require constant nursing care and attention), and a score of 6 indicates death. Percentages might not total 100 because of rounding. mRS, modified Rankin Scale.

Overall, 157 patients (63.8%) achieved a good functional outcome after 90 days. Patients treated primarily with the SOFIA PLUS achieved a good functional outcome in 123 cases (62.1%), while there were 34 patients (70.8%) with SOFIA as frontline device who had a good outcome (*p* = 0.316). Figure 2 shows the distribution of mRS values after 90 days for available data (*n* = 212) as well for the dataset after imputation (*n* = 246) to illustrate possible implications of data imputation on the primary outcome.

### Secondary outcomes

After first-line therapy using SOFIA catheters for aspiration alone, successful recanalization (mTICI ≥2b) was achieved in 72.8% (179/246) of patients (mTICI ≥ 2c in 57.3%, 141/246; mTICI = 3 in 34.1%, 84/246) after 33.4 ± 24.6 min. The first pass effect to attain mTICI 2c or 3 was 48.8% overall, with no differences between SOFIA and SOFIA PLUS. A mean of 1.4 aspiration maneuvers was performed overall. In *n* = 47 (19.1%), stent-retrievers were used as a second-line therapy, resulting in an overall complete recanalization in 91.5% (225/246) of cases (mTICI ≥ 2c in 191/246, 77.6%; mTICI = 3 in 118/246, 47.9%) after overall 38.73 ± 27.3 min.

After the procedure, the median NIHSS at 24 h was 4 (2–9), and follow-up imaging demonstrated an ASPECTS of 7 (5–8) with a mean delta ASPECTS of 1.64 ± 2.10. Patients were discharged with a median NIHSS of 2 (0–5) and a mRS of 2 (1–4). After 90 days, patients presented with a median mRS of 1 (0–3), with a mortality rate of 24.4%.

A statistical comparison of periprocedural and outcome parameters associated with usage of SOFIA and SOFIA PLUS can be found in Tables 2, 3.

**Table 2:** Periprocedural parameters in descriptive statistics for all enrolled patients, along with comparative statistics for patient cohorts using SOFIA and SOFIA 6F PLUS.

	Complete cohort	6F (*n* = 198)	5F (*n* = 48)	*p* value
Time from puncture to first recanalization	21.6 + − 13.1	22.1 + − 13.8	19.4 + − 9.4	0.965
Time from onset to final recanalization	309.3 + − 204.1	320.6 + − 219.4	262.9 + − 111.9	0.011
Procedure duration	36.5 + − 28.0	37.6 + − 28.9	32.3 + − 23.7	0.117
Aspiration maneuvers	1.42 ± 0.85	1.45 ± 0.89	1.27 ± 0.64	0.116
mTICI ≥2b after first line (only aspiration, %)	72.8	71.2	79.2	0.874
mTICI ≥2b at the end of procedure (%)	91.5	90.1	93.8	0.597
mTICI ≥2c after first line (only aspiration, %)	57.3	56.6	60.4	0.698
mTICI ≥2c at the end of procedure (%)	77.6	76.8	81.3	0.659
mTICI 3 after first line (only aspiration, %)	34.1	35.4	29.2	0.495
mTICI 3 at the end of procedure (%)	47.9	47.5	50.0	0.872
First pass effect (%)	48.8	48.0	52.1	0.614
Embolization in new territory	9 (3.7)	9 (4.5)	0	0.214

Metrics are listed as median (IQR) or mean ± standard deviation for continuous variables, and number (%) for ordinal variables. mTICI, Modified treatment in cerebral ischemia score. First pass effect was defined as reaching mTICI 2c or 3 after the first maneuver.

**Table 3:** Early and late outcome parameters in descriptive statistics for all enrolled patients, along with comparative statistics for patient cohorts using SOFIA and SOFIA 6F PLUS.

	Complete cohort	6F (*n* = 198)	5F (*n* = 48)	*p* value
NIHSS at 24 h	4 (2–9)	5 (2–11)	2 (1–7)	0.013
ASPECTS at day-1 imaging	7 (5–8)	7 (5–8)	7 (5–9)	0.028
Any ICH at day-1 imaging	80 (32.5)	71 (36.4)	9 (19.2)	0.321
Symptomatic ICH	11 (4.4)	10 (5.1)	1 (2.1)	0.257
NIHSS at discharge	2 (0–5)	2 (1–5)	1 (0–4)	0.159
mRS at discharge	2 (1–4)	2 (1–4)	2 (1–3)	0.192
mRS after 90 days	1 (0–4)	1 (0–3)	2 (1–4)	<0.001

Metrics are listed as median (IQR) or mean ± standard deviation for continuous variables, and number (%) for ordinal variables. NIHSS, National Institute of Health Stroke Scale; ASPECTS, Alberta Stroke Program Early Computed Tomography Scores; ICH, intracerebral hemorrhage; mRS, modified Rankin Scale score.

### Safety outcomes and complications

Overall, no unexpected procedural complications occurred, as adjudicated by the independent CEC. No dissection, perforations or severe vasospasm were recorded as well as no device-related complications. A total of nine cases in the study exhibited embolization into a new or previously unaffected vascular territory (ENT). This phenomenon was primarily observed in patients with proximal occlusions (eight out of nine cases). Three procedure-related events were observed (two cases of groin hematoma and one case of pseudoaneurysm of the femoral artery).

Within the study cohort, a total of 80 patients (32.5%) experienced any ICH. The majority of these hemorrhages were classified as subarachnoid hemorrhages or scattered or confluent petechiae without significant mass effect, accounting for 63.8% of all hemorrhages. Eleven patients (4.5%) did exhibit large hemorrhages accompanied by extensive mass effect within the infarcted brain, of which four were adjudicated to be the cause of a fatal outcome. Overall, hemorrhages were classified according to HBC as follows: 12 (4.9%) type 1a, 16 (6.5%) type 1b, 16 (6.5%) 1c, 9 (3.7%) type 2, 2 (0.8%) type 3a, 3 (1.2%) type 3b, 11 (4.5%) type 3c, and 1 (0.4%) type 3d.

Table 4 summarizes the primary and secondary outcomes of the study protocol.

**Table 4:** Overview of all primary and secondary outcomes of the study protocol.

Outcomes	No./Total (%)
Primary efficacy outcome good functional outcome (mRS 90 ≤ 2)	157/246 (63.8)*
Secondary outcomes ≥ mTICI2b after primary aspiration	179/246 (72.8)
≥ mTICI2b after usage of an additional device	225/246 (91.5)
Device-related adverse events	0
Procedure-related adverse events	3/246 (1.2)
Embolization in new territory	9/246 (3.7)
Vessel perforation or dissection	0
Symptomatic intracranial hemorrhage	11/246 (4.4)

mRS, modified Rankin Scale score; mTICI, Modified treatment in cerebral ischemia score. *We utilized multiple imputation (13.8%, *n* = 34/246) to address missing clinical data. The “missForest” R package, designed for mixed-type and non-normally distributed data, was employed for data imputation. This method employs a random forest model trained on observed values to predict missing ones. In a worst-case scenario analysis, assuming all missing mRS 90 values as 6, 58.9% (*n* = 145/246) of patients would have achieved a favorable clinical outcome (mRS 0–2).

To create a vacuum for the aspiration maneuver, a syringe was used in 56.5% of patients, a vacuum pump in 50% of cases and both in 11.3%. No differences in attaining FPE or good clinical outcome were conceived when comparing groups with aspiration using a syringe as compared to vacuum pump usage (*p* = 0.9609 and *p* = 0.1813, respectively).

In a minority of cases, a balloon guiding catheter was used (18/246, 7.3%).

There were protocol violations concerning missed time windows for endpoint evaluation, as well as a result from violation of inclusion and exclusion criteria, leading to treatment beyond 6 h of symptom onset in 11 patients. Three-month follow-up was missing in 34 patients. Further details can be seen in the flowchart diagram of the inclusion process (Figure 1).

## Discussion

In this prospective, multi-center study of 14 participating European centers, we evaluated the safety and efficacy of the SOFIA/SOFIA PLUS catheter for direct aspiration as a first-line treatment technique (SESAME) of LVO in the anterior circulation in 246 patients. Our results demonstrated that 63.8% of patients achieved a favorable outcome, using a direct contact aspiration technique with SOFIA or SOFIA PLUS as a first-line treatment strategy.

These results are in the upper spectrum of comparable randomized controlled trials (RCT) that have been published previously. Lapergue et al. randomized patients in the ASTER trial to primary treatment with stent-retrievers or direct contact aspiration and reported a rate of 45.3% of patients with functional independence after 90 days in their cohort treated with contact aspiration (7). Similar results were reported in the COMPASS trial with 52.0% and in the PROMISE trial with 61.0% in patients who were treated using contact aspiration (8, 18).

In our study, the use of aspiration catheters alone resulted in a rapid successful recanalization in a large proportion (72.8% mTICI ≥2b) of patients. After the additional use of a stent-retriever (in 19.1% of cases), this rate increased to a 91.5% of mTICI ≥2b.

These findings are again in line with previously published data, such as the ASTER RCT where 63.0% of patients achieved complete recanalization (mTICI ≥ 2b) with direct contact aspiration as the initial modality, increasing to 84.9% after stent-retriever usage in 32.8% of cases. Similarly, the COMPASS RCT study reported 83.0% complete recanalization (mTICI ≥ 2b) with the first modality, which improved to 92.1% after stent-retriever usage in 21.2% of cases. The PROMISE registry demonstrated 70.6% initial complete recanalization (mTICI ≥ 2b) using the PENUMBRA ACE68/64 as the primary device, and 91.1% final TICI score after using additional stent-retriever devices in 20.9% of cases. These consistent results across studies highlight the efficacy of the contact aspiration technique with aspiration catheters and the potential benefit of incorporating stent-retrievers in selected cases to further improve recanalization rates.

Our study provides evidence of a favorable safety profile associated with the use of the SOFIA aspiration catheters. We observed low rates of symptomatic ICH in follow-up imaging in only 4.4% of patients, while any ICH was perceived in 32.5% of cases. While the number of any detected ICH may appear high at first glance, our figures are also within the range of comparable studies (COMPASS 46.3%, ASTER 36%, PROMISE 34%), and largely reflect the occurrence of subarachnoid or circumscribed petechial hemorrhages (19), the presence of which is less likely to have a significant impact on the clinical outcome. Still, 27 patients overall had parenchymal hemorrhages. Ultimately, the rate of symptomatic hemorrhage with 4.4% was similar to large stroke registries such as HERMES or STRATIS with 4.4 and 2.5%, respectively (3, 20). Of note, there was a tendency toward a lower risk of symptomatic ICH with direct aspiration, as the alleged endothelial damage seems to be lower compared with stent retrievers, especially with repeated maneuvers (21).

Additionally, the rates of ENT amounted to 3.7% being relatively low in our study. In a study of 259 patients using only stent retrievers, Kaesmacher et al. generally found more frequent rates of ENT—but more sensitive diffusion-weighted MRI was performed as a control imaging, with severe cases of ENT observed in 5% (22). In an analysis of the ESCAPE-NA1 trial, ENT was observed in 9.3% of cases in a cohort of 1,092 patients (23). Thereby, our data do not confirm the hypothesized concern of increased thrombus spread or fragmentation with a primary contact aspiration as compared to an approach using a stent-retriever (24).

The majority of clinical adverse events reported were consistent with those commonly observed in patients with severe stroke. These included complications such as infection, pneumonia, and new stroke. These events can be attributed to the patients’ underlying condition and the associated comorbidities commonly seen in this population. Among the unexpected events, the majority were related to the worsening of pre-existing or newly diagnosed cancer, and therefore not directly associated with the procedure or the device but rather to the underlying health status of the patients.

Overall, there were several significant differences in patient parameters when comparing patients that where primarily treated with either SOFIA or SOFIA PLUS, such as stroke severity measured by NIHSS after 24 h or the rate of patients with functional independence after 90 days. These differences can be attributed to the different purposes of the catheters and an ensuing selection bias. The SOFIA PLUS with its larger inner lumen is better suited for large thrombi by contact aspiration in proximal large vessel occlusions such as the distal ICA, the carotid T or the M1 segment. Patients with such proximal occlusions have in general more severe symptoms and are at higher risk of a poor functional outcome due to the larger area at risk. Patients with medium or distal vessel occlusions, on the other hand, were predominantly treated with SOFIA catheters, which, due to its smaller outer lumen, can access the site of vessel occlusion more easily. When used as intended, there does not appear to be an overall increased risk of vascular complications associated with the use of SOFIA PLUS according to our results. In accordance with its intended application for distal occlusions, employing the 5F SOFIA resulted in a higher proportion of patients achieving favorable clinical outcomes.

When considering the properties of the SOFIA catheters for thrombectomy procedures, several notable features distinguish it from other catheters in its class. Firstly, the SOFIA catheters offers the advantage of navigability without the need for a triaxial system, as demonstrated by the SNAKE technique (25). This characteristic enhances the procedural speed by eliminating the requirement for additional equipment, streamlining the workflow for clinicians.

Another significant property of the SOFIA catheters is its ability to be steam-shaped, a feature not commonly found in many large-bore catheters according to their Instructions for Use (IFU). This attribute has been lauded by numerous authors for its efficacy in facilitating the crossing of delicate anatomical structures such as the ophthalmic artery. Consequently, the necessity for supplementary systems like the Wedge catheter is nearly obviated, potentially reducing procedural complexity and associated risks.

Additionally, SOFIA catheters stand out by their shaft’s unique capability to be torqued to facilitate distal navigation (26). This feature, to the best of our knowledge, is unparalleled among similar devices and has been observed to greatly enhance distal navigation during procedures. This enhanced maneuverability may significantly augment the safety profile and overall success rates of thrombectomy interventions, underscoring the clinical significance of this attribute.

Despite the promising results of our study, there are several limitations that should be considered. First, the study design was a single-arm, observational registry study, which may introduce inherent selection biases and limit the generalizability of the findings. The absence of a control group makes it difficult to directly compare the outcomes with alternative treatment approaches. Second, the study included patients from multiple centers, which may introduce variability in treatment protocols, operator expertise, and patient selection criteria. Third, the follow-up period of 90 days may not capture all long-term outcomes and potential complications that may arise later. In that respect it should be noted that there was a considerable rate of loss to follow-up at 90 days of 13.8% (*n* = 34/246). Multiple imputation as the method of choice was used to supplement missing clinical endpoints. In a worst-case scenario analysis, assuming all missing mRS 90 values as 6, 58.9% (*n* = 145/246) of patients would have achieved a favorable clinical outcome (mRS 0–2). Patients with a stroke onset of more than 6 h and a pre-stroke mRS of more than 1 were not included in the study, so we cannot confidently transfer our results to these patient populations. Patients with isolated A1/A2 occlusions were not actively excluded from the study. However, it appears that these cases were less frequently included by treating physicians. The challenges associated with the direct aspiration of A1/A2 occlusions, due to their technical difficulty and the perception that they may be less amenable to primary aspiration thrombectomy, likely influenced this selection bias. Despite this, such occlusions were intended to be part of the study cohort. This bias should be noted as another limitation, given that A1/A2 occlusions were within the eligibility criteria.

In conclusion, our prospective, multicentric SESAME study supports the SOFIA catheters as highly effective and safe tools for direct aspiration in the treatment of acute anterior ischemic stroke. Our results demonstrate excellent rates of recanalization and favorable functional outcomes, reaffirming the clinical benefits of this technique. These findings, combined with the positive outcomes reported in other studies, support the application of the SOFIA/SOFIA PLUS catheter in the treatment of large vessel occlusions in clinical practice.

## Data Availability

The data that support the findings of this study are available from the corresponding author, MM, upon reasonable request. Requests to access the datasets should be directed to markus.moehlenbruch@med.uni-heidelberg.de.
