# Supplementary material for: Safety and effectiveness of SOFIA/SOFIA PLUS for direct aspiration as first line treatment in patients with acute anterior ischemic stroke: results from the prospective, multicentric SESAME study
Source: Front Neurol. 2024 Sep 25;15:1441810. doi: 10.3389/fneur.2024.1441810 (PMC11461265; doi:10.3389/fneur.2024.1441810)
Supplement: Supplementary file 1 [file Data_Sheet_1.PDF]

SESAME - Safety and Effectiveness of SOFIA™/SOFIA™ PLUS when used for direct aspiration as a first line treatment technique in patients suffering an Acute Ischemic Stroke in the anterior circulation.

Title:	Safety and Effectiveness of SOFIA™/SOFIA™ PLUS when used for direct aspiration as a first line treatment technique in patients suffering an Acute Ischemic Stroke in the anterior circulation.
Protocol Number:	
Device Class/ Study Phase:	Class III/Post Marketing study
Test Device:	SOFIA™, SOFIA™ PLUS
International study coordinator :	Dr. Markus Möhlenbruch<br>Head of Interventional Neuroradiology<br>Neurological Clinic<br>Department of Neuroradiology<br>Im Neuenheimer Feld 400<br>69120 Heidelberg<br>Germany
Imaging Core Lab:	Prof. Dr. med. Fiehler<br>Eppdata GmbH<br>Locksteder Steindamm 18<br>22529 Hamburg<br>Germany
Statistics	Dr. Susanne Bonekamp<br>Neurological Clinic<br>Department of Neuroradiology<br>Im Neuenheimer Feld 400<br>69120 Heidelberg<br>Germany
Protocol Version and Release Date:	V1.7<br>July 2019
Revision History:	N/A

THIS PROTOCOL CONTAINS CONFIDENTIAL INFORMATION AND SHALL ONLY BE DISCLOSED TO INVESTIGATORS AND AUTHORITIES IN CHARGE OF THE REVIEW. CONFIDENTIAL INFORMATION SHALL NOT BE REPRODUCED WITHOUT THE PRIOR WRITTEN AGREEMENT OF THE STUDY COORDINATOR.

STATEMENT OF THE INVESTIGATOR

- I agree to conduct the study in accordance with this protocol and to make no changes except when necessary to protect the safety, rights or welfare of patients. If such a change occurs, I will promptly inform the Study Coordinator of this event.
- I agree to personally conduct or supervise the study, and that all associates, colleagues and employees assisting me in the conduct of this study are informed of their obligations in meeting study commitments.
- I have read and understand the information in the Investigator's Brochure and/or the Instructions for Use, including the potential risks, expected adverse events and potential side effects of the study itself and the product being studied.
- I agree to protect the rights of my patients and obtain informed consent from those who may participate in this study, in accordance with the ISO14155 and Helsinki declaration, the EU Data Protection Regulation, and the local regulation and the requirements of my Institutional Review Board/Ethics Committee.
- I agree to report to the Study Coordinator adverse experiences that occur in the course of this study, in accordance with the study protocol requirements.
- I agree to maintain adequate and accurate records in accordance with study requirements and those records will be made available for inspection by the Study Coordinator, IRB/EC or regulatory bodies such as BfArm, ANSM etc..
- I agree to provide my Institutional Review Board/Ethics Committee with all the information required to support both the initial and continuing review and approval of this study and I will not implement this study until such approval has been obtained. I agree to promptly report to the Institutional Review Board/Ethics Committee all changes in research activity and all unanticipated problems involving risk to patients or others.
- I agree to comply with all other requirements regarding the obligations of clinical investigators, as outlined in 21 CFR Part 812.100-150, ISO 14155 and local regulation. A copy of those regulations has been provided to me.

____
Investigator Name, PRINT

______________________________ ________________
Investigator SIGNATURE Date

____
Institution Name

1 Content

1 Content ... 3
2 Study Overview ... 6
2.1. Summary Data Collection and Schedule of Events ... 10
3 Introduction ... 11
4 Study Overview ... 12
4.1. Study Design ... 12
4.2. Endpoints ... 13
4.2.1. The primary endpoint ... 13
4.2.2. The secondary endpoints ... 13
4.2.3. Supplemental Endpoints ... 14
4.3. Study Population ... 14
4.4. Rationale ... 15
4.5. Device Description / Principles of Operation ... 15
4.5.1. Device Description ... 15
5 Study Procedures ... 16
5.1. Screening ... 16
5.2. Informed Consent ... 17
5.3. Pre-treatment Assessment ... 17
5.4. Enrollment ... 18
5.5. Procedure ... 18
5.6. Additional Devices or Procedures ... 19
5.7. Angiographic Imaging ... 19
5.8. Follow-up at 24 ± 12 Hours Post-procedure ... 19
5.9. Follow-up at Discharge (7±3Days Post-procedure) ... 20
5.10. Follow-up at 90 ± 14 Days Post-procedure ... 20
5.11. Unscheduled Visits ... 21
5.12. Termination of Patient Participation ... 21
6 Assessment of Safety and Effectiveness ... 21

6.1. Safety.....21

6.1.1. Device Deficiency.....22

6.1.2. Adverse Events and Serious Adverse Events.....22

6.2. Effectiveness.....24

6.2.1. Functional Outcome.....24

6.2.2. Angiographic Outcome.....24

6.3. Risk/Benefit Analysis.....25

6.3.1. Angiography.....25

6.3.2. Computed Tomography Scan (CT Scan) and Magnetic Resonance Imaging (MRI).....25

6.3.3. Thrombectomy/Embolectomy.....25

7 Imaging Core Lab.....26

8 Data Management.....27

8.1. Data Prottection.....27

9 Statistical Methods.....27

9.1. Sample Size Justification.....27

9.2. Statistical Analysis.....28

9.2.1. Definition of Analysis Samples.....28

9.2.2. Effectiveness.....28

9.2.3. Safety.....29

9.2.4. Recanalization:.....29

9.2.5. Clinical endpoints / quality of life:.....29

9.2.6. Supplemental Endpoints.....30

9.2.7. Analysis of Adverse Events:.....30

9.2.8. Interim Analysis and Early Stopping Rules.....30

10 Quality Control and Quality Assurance.....30

10.1. Training of Investigator and Site Personnel.....30

10.2. Data Monitoring.....31

10.2.1. Site Qualification.....31

10.2.2. Site Initiation Visit.....31

10.2.3. Data Monitoring Site Visit 31
10.2.4. Study Close-Out Site Visit 32
10.3. Investigator Requirements 32
10.3.1. Ethics and Regulatory Considerations 32
10.3.2. Protocol Amendment and Modifications 37
10.4. Clinical Events Committee 37
10.5. Corelab Imaging Review 38
11 Appendix 38
11.1. NIHSS Form 38
11.2. mRS Definitions 38
12 Bibliographie 39

2 Study Overview

Study Devices:	SOFIA™, SOFIA™ PLUS
Title:	Safety and Effectiveness of SOFIA™/SOFIA™ PLUS when used for direct aspiration as a first line treatment technique in patients suffering an Acute Ischemic Stroke in the anterior circulation. (SESAME)
International study coordinator	Dr. Markus Möhlenbruch<br>Head of Interventional Neuroradiology<br>Neurological Clinic<br>Department of Neuroradiology<br>Im Neuenheimer Feld 400<br>69120 Heidelberg<br>Germany
Device Description:	The SOFIA™/ SOFIA™ PLUS Catheter is a non-tapered, single-lumen, flexible catheter equipped with the coil and the braid reinforcement. The distal segment is steam-shapeable to facilitate vessel selection and also has a hydrophilic coating for navigation through the vasculatures. The radiopaque marker is located at the distal end of the catheter for visualization under fluoroscopy. All devices received CE-mark and are used according to the Instructions For Use.
Study Design:	European, multi-center, single arm, prospective, observational registry.
Study Purpose:	To demonstrate that use of SOFIA™/SOFIA™ PLUS catheter for direct aspiration as a first line treatment technique is fast, safe and effective in patients suffering an Acute Ischemic Stroke when assessed at 24 hours, discharge and 90 days after treatment.
Sample Size & Duration of Study:	250 patients will be enrolled. All patients will be followed for 90 days or until death.
Number of Sites:	Up to 20 European sites (France, Germany, Austria, Italy, Netherlands)
Allocation:	Non-randomised, single arm
Patient Population:	Patients who are at least 18 years of age presenting with an acute ischemic event in the anterior cerebral circulation that can be treated within 6 hours from AIS symptom onset. Those eligible to be treated with SOFIA™/ SOFIA™ PLUS will be enrolled after having signed an informed consent form (or having one signed on his or her behalf by a legally authorized

	representative or an independent physician).
Patient Selection Criteria:	Patient selection criteria are established according to clinical, neurological and imaging considerations. Patients who meet all of the inclusion criteria and none of the exclusion criteria will be eligible for study participation.
Target Enrollment Period & Study Duration:	An 42 month enrollment period is estimated. This period will begin upon enrollment of the first patient and end upon enrollment of the last patient into the study.<br>Total study duration is estimated to be up to 45 months (up to 42 months of enrollment + 3 months of follow-up).
Inclusion Criteria:	1. Participant is ≥ 18<br>2. Demonstrated occlusion of the distal intracranial carotid artery, middle cerebral artery (M1 or M2) or anterior cerebral artery (A1 or A2) proven by CT and/or MRI<br>3. NIHSS ≥ 2 and ≤ 30 at screening<br>4. Start of the thrombectomy procedure within 6 hours of the onset of stroke symptoms<br>5. Pre event mRS ≤1<br>6. Informed consent by the patient, legal guardian, or inclusion of patient under presumptive will after consultation of an independent physician and statement of investigator
Exclusion Criteria:	1. Patient is more than 6 hours from symptom onset<br>2. Rapidly improving neurologic examination<br>3. Evidence of cerebral ischemia in the posterior circulation<br>4. Severe unilateral or bilateral carotid artery stenosis requiring stent treatment<br>5. Presence of an existing or pre-existing large territory infarction<br>6. Absent femoral pulses<br>7. Excessive vascular tortuosity that will likely result in unstable access<br>8. Pregnancy; if a woman is of child-bearing potential a urine or serum beta HCG test is positive<br>9. Known contrast product allergy<br>10. Patient has a severe or fatal comorbidity that will likely prevent improvement or follow up or that will render the procedure unlikely to benefit the patient

	11. Evidence of intracranial hemorrhage (SAH, ICH, etc.)<br>• Imaging exclusion criteria:<br>• Significant mass effect with midline shift or intracranial tumor<br>• Baseline non-contrast CT or DWI MRI evidence of a moderate/large core defined as extensive early ischemic changes of Alberta Stroke Program Early CT score (ASPECTS) 0-5
Visit Schedule and Assessments:	*Screening:*<br>Informed consent form<br>Demographic information<br>Inclusion/exclusion criteria<br>Examination and vital signs<br>Medical history and concomitant medication<br>NIHSS<br>Pre event mRS<br>MRI and/or CT<br>rtPA status<br>*Procedure:*<br>Sedation<br>Anticoagulation and antiplatelet therapy<br>DSA (pre-procedure, procedure, post procedure)<br>Additional and adjunctive devices or therapy (as needed)<br>*24h:*<br>NIHSS, mRS, MRI and/or CT<br>*Discharge:*<br>NIHSS, mRS<br>*90 days:*<br>mRS, Quality of Life Questionnaire
Analysis Population:	Per protocol (primary and secondary endpoints)
Primary Endpoint:	Clinical outcome defined as mRS ≤ 2 at 90 days (assessment performed by an independent neurologist)
Secondary Endpoints:	The secondary endpoints will consist of:<br>Safety endpoints:<br>• Occurrence of major neurological events (stroke, intracranial hemorrhage, intracerebral hemorrhage, etc.) prior to discharge<br>• Devices and procedure related adverse events within 90 days of fol-

	low up<br>• Occurrence of embolization in previously uninvolved (or new) territories (ENT) as seen on the final control angiogram at the end of the procedure<br>• Occurrence of symptomatic intracranial hemorrhage (sICH) within 24 hours<br>• Occurrence of intracranial vessel damage at the end of the procedure.<br>Recanalization:<br>• Proportion of patients having complete recanalization (TICI≥ 2b) just after first line aspiration treatment*<br>• Proportion of patients having complete recanalization (TICI ≥ 2b) after thrombectomy using an additional device*<br>• Time from groin puncture to complete recanalization (TICI ≥ 2b) in patients after first line aspiration treatment<br>• Time from groin puncture to complete recanalization (TICI ≥ 2b) after thrombectomy using an additional device<br>• Time from CT-scan/MRI at the institution to groin access<br>• Time from symptom onset to CT-scan/MRI<br>Clinical endpoints / quality of life:<br>• NIHSS score at 24 hours<br>• NIHSS score at discharge<br>• mRS at discharge<br>• Quality of Life at 90 days<br>* assessed by an imaging core lab
Supplemental endpoints	Imaging:<br>• Difference of ASPECT scores in CT/MRI pretreatment vs. 24h<br>• In the subgroup of patients with additional perfusion CT (as per local standard of care): volume of saved brain tissue determined by predictive modeling<br>Health Economics:<br>• Device costs (standardized cost of all devices as well as human resources and medication used during index procedure)<br>• Hospital length of stay
Intended Use Statement:	The SOFIA™ Catheter is intended for use in removal/aspiration of emboli and thrombi from selected blood vessels in the arterial system, including the peripheral and neurovasculatures.

Statistical Methodology:	All patients included and treated with SOFIA™ / SOFIA™ PLUS during the study will be analyzed (PP). Categorical variables will be described by their frequency distribution and ranges bilateral 95% confidence. Continuous variables will be described by their average, minimum standard deviation, maximum, median and quartiles. All endpoints will be evaluated for SOFIA™ and SOFIA™ Plus catheters separately.<br>mRS at 3 month ≤ 2 (yes / no) will be described by their distribution frequencies and intervals bilateral associated 95% confidence.<br>Statistical test will be performed with a type I error risk of 5%.<br>The rate of events for which a date of onset has been collected will be described by a survival curve according to Kaplan-Meier method and the associated Kaplan-Meier estimators will be calculated.<br>An interim analysis will be performed after 50 patients are enrolled.
Anticipated Timelines	FPI *October 2017*<br>LPI *April 2021*<br>FPO *January 2018*<br>LPO *July 2021*<br>Database lock *September 2021*
Financing	Financial support for the SESAME study will be pro MicroVention Europe, a French limited liability company, with capital of 40 000€, registered with the Registry of Commerce and Companies in Versailles under number 440 775 674 RCS, with registered address at 30 bis, rue du Vieil Abreuvoir, 78100 Saint-Germain en Laye, France.

2.1. Summary Data Collection and Schedule of Events

Data will be collected on electronic case report forms at the pre-procedure, index procedure and post-procedure visits. A schedule of the study activities by visit is provided below.

Table 1 Schedule of Study Activities by Visit:

Visit	Selection/ Inclusion	Procedure	24h	Discharge	3 months FU	Unscheduled visit[2]
Patient Demographic	x					
Patient informed consent	x					

Incl/excl criteria	x					
Clinical status (mRS[3], NIHSS[4])	x		x	X	x	X
Quality of Life					x	x
Medical history	x					
Relevant concomitant medication	x					X
Procedure		x				
Antiplatelet and antico-agulant therapy[1]		x				
Procedure complications		x				
Procedure results		x				
AE(s)		x	x	X	x	X
CT/MRI	x		x			X

NOTES:

1 To be administered just before procedure
2 Any data collected based on the reason for unscheduled visit
3 Performed by an independent neurologist
4 Selection, 24h, Discharge

3 Introduction

Acute ischemic stroke (AIS) remains the leading cause of long-term disability in the United States with approximately 795,000 individuals afflicted in the United States every year.[1] Direct medical costs of stroke are upwards of $17 billion, and the cost in terms of human suffering remains high, with more than 50% of stroke patients requiring discharge to a rehabilitation or skilled nursing facility. Up to one half of AIS is related to emergent large vessel occlusion, most commonly of the internal carotid artery (ICA) or middle cerebral artery (MCA). Until recently, intravenous tissue plasminogen activator given within 4.5 hours of symptom onset was the only treatment approved by the US Food and Drug Administration, and early randomized trials of endovascular stroke therapy failed to demonstrate benefit. More recently, however, multiple trials have now unequivocally established the clinical benefit associated with mechanical thrombectomy for certain well-

selected patients. The improved results observed in these trials were due to both increased understanding of patient selection as well as the use of newer, more effective treatment devices.[1-8]

Early recanalization of the occluded artery leads to improved clinical outcomes in patients with AIS, most likely through protection of the penumbra. If timely recanalization can be achieved and reperfusion established, damage to the penumbra region may be prevented, thus resulting in improved neurological outcome with less deficits and a reduction in stroke-related mortality and morbidity. [4, 9-15]

Recently, first-line, direct-aspiration first-pass technique performed with new thrombectomy devices generated promising results in several retrospective studies. This distal suction system, with a high level of endovascular navigability, provided high recanalization rates (up to 97%) with low morbidity and good functional outcomes.[12, 16-20] Patients treated with first-line, direct-aspiration first-pass technique versus mechanical thrombectomy using a stent retriever achieved higher final recanalization rates but adjunctive devices or rescue procedures had to be used more frequently [20].

Our research aims to show that a first line strategy of recanalization by thrombectomy using a distal suction system provides fast recanalization by sole aspiration with good safety and Effectiveness. Thus, the primary aim of this study is to assess the safety and effectiveness of the SOFIA™/SOFIA™ PLUS catheter for direct aspiration as a first line treatment technique (SESAME) in patients with acute ischemic stroke from large vessel occlusion.

4 Study Overview

4.1. Study Design

This is a multi-center, single arm, prospective, observational registry of the SOFIA™/ SOFIA™ PLUS Catheter in Europe. Consecutive patients presenting within 6 hours of symptom onset with an anterior circulation large vessel occlusion (LVO) acute ischemic stroke (within the internal carotid artery and internal carotid terminus, middle cerebral –M1/M2 segments) will be treated using aspiration thrombectomy as first intention and site routine practice. Devices received CE-mark and will be used according to the 'Instructions For Use'.

The follow-up visits will occur at 24 +/- 12 hours, at patient discharge, and 90+/-14 days post-procedure.

Furthermore the study design is adaptive, prospectively stating interim analyses with specified stopping rules, which allow for the possibility of the study to terminate early based on either a determination of study success or of the futility to continue further enrollment.

4.2. Endpoints

The primary objective of this study is to observe the percentage of good clinical outcomes defined as ≤2 at 90 days.

4.2.1. The primary endpoint

Primary endpoint of the study will be mRS ≤ 2 at 90 days as assessed by an independent neurologist)

4.2.2. The secondary endpoints

Safety endpoints:

- The occurrence of major neurological events (stroke, intracranial hemorrhage, intracerebral hemorrhage, etc.) prior to discharge
- Devices and procedure related adverse events within 90 days of follow up
- Occurrence of embolization in previously uninvolved (or new) territories (ENT) as seen on the final control angiogram at the end of the procedure
- Occurrence of symptomatic intracranial hemorrhages (sICH) within 24 hours
- Occurrence of intracranial vessel damages at the end of the procedure.

Recanalization:

- Proportion of patients having complete recanalization (TICI ≥ 2b) just after first line aspiration treatment compared to historic controls based on published RCTs*
- Proportion of patients having complete recanalization (TICI ≥ 2b) after thrombectomy using an additional device
- Time from groin puncture to complete recanalization (TICI ≥ 2b) in patients after first line aspiration treatment (SESAME).
- Time from groin puncture to recanalization (TICI ≥ 2b) after thrombectomy using an additional device
- Time from CT-scan/MRI at the institution to groin access
- Time from symptom onset to CT-scan/MRI

* assessed by an imaging core lab.

Clinical endpoints / quality of life:

- NIHSS score at 24 hours
- NIHSS score at discharge
- Quality of Life questionnaire at 90 days

4.2.3. Supplemental Endpoints

Imaging endpoints:

- Difference of ASPECT scores in CT/MRI pretreatment vs. 24h

In the subgroup of patients with additional perfusion CT (as per local standard of care):

- Volume of saved brain tissue determined by predictive modeling

Health Economics:

- Device costs (standardized cost of all devices as well as human resources and medication used during index procedure)
- Hospital length of stay

4.3. Study Population

Up to 250 consecutive patients at up to 15 centers in Europe with symptoms of acute ischemic stroke who are eligible for inclusion into this study. The patients must meet the following inclusion criteria and none of the exclusion criteria:

Inclusion Criteria:

- Participant is ≥ 18
- Demonstrated occlusion of the distal intracranial carotid artery, middle cerebral artery (M1 or M2) or anterior cerebral artery (A1 or A2) in the anterior circulation proven by CT and/or MRI
- NIHSS ≥ 2 and < 30 at screening
- Start of the thrombectomy procedure within 6 hours of the onset of stroke symptoms
- Pre event mRS ≤ 1
- Patient or patient's legally authorized representative or impartial witness/independent physician has received information about data collection and has signed and dated an Informed Consent Form

Exclusion Criteria:

- Patient is more than 6 hours from symptom onset
- Evidence of cerebral ischemia in the posterior circulation
- Rapidly improving neurologic examination
- Severe unilateral or bilateral carotid artery stenosis requiring stent treatment
- Absent femoral pulses
- Presence of an existing or preexisting large territory infarction
- Excessive vascular tortuosity that will likely result in unstable access

- Pregnancy; if a woman is of child-bearing potential and urine or serum beta HCG test is positive
- Known contrast product allergy
- Patient has a severe or fatal comorbidity that will likely prevent improvement or follow-up or that will render the procedure unlikely to benefit the patient
- Evidence of intracranial hemorrhage (SAH, ICH, etc.)

Imaging exclusion criteria:

- Significant mass effect with midline shift or intracranial tumor
- Baseline non-contrast CT or DWI MRI evidence of a moderate/large core defined as extensive early ischemic changes of Alberta Stroke Program Early CT score (ASPECTS) 0-5

4.4. Rationale

Several publications describing the use of aspiration as a first line treatment technique in AIS patients have shown superior technical results with similar clinical outcomes to those seen when using a traditional stent retriever. They have also shown decreased procedure time and cost. The aim of this study is to show similar results in terms of speed, Effectiveness and safety when SOFIA™/ SOFIA™ PLUS is the catheter used for first line aspiration thrombectomy.

4.5. Device Description / Principles of Operation

A detailed description of the SOFIA™/ SOFIA™ PLUS catheter is contained in the 'Instructions For Use' for these devices, which have obtained commercial authorization in Europe (CE Mark). Described herein are brief descriptions of the System and the principles of operation.
The SOFIA™/SOFIA™ Plus Catheter is intended for use in removal/aspiration of emboli and thrombi from selected blood vessels in the arterial system in patients with AIS, including the peripheral and neurovasculatures.

4.5.1. Device Description

The SOFIA™ (Soft Torqueable Catheter Optimized For Intracranial Access) and SOFIA™ PLUS Catheter are single lumen, flexible catheters, designed with coil and braid reinforcement. The SOFIA™ / SOFIA™ PLUS catheters have a soft distal tip for easy navigation in tortuous vessels. The tip is steam shapable and the proximal shaft torquable to help steer around challenging bifurcations. The coil and braid construction provides enhanced kink resistance and 1:1 push / pull control. Once navigated to the site of the occlusion, the SOFIA™ / SOFIA™ PLUS catheters can be used in conjunction with an aspiration source, such as a pump or syringe, to facilitate aspiration thrombectomy of the occluded vessel. The SOFIA™ / SOFIA™ PLUS catheters have large lumens, developed to maximize aspiration power and capture of thrombus.

SOFIA™ & SOFIA™ PLUS key dimensions:

Dimension	SOFIA™	SOFIA™ PLUS
French size	5F	6F
Outer diameter	.068“	.0835“
Inner diameter	.055“	.070“
Working lengths	115cm<br>125cm	115cm<br>125cm<br>131cm
Compatibility	6F sheaths<br>Guide catheters ≥.070“	6F sheaths<br>BGC ID ≥.085“

Table 2: Features of the SOFIA™ /SOFIA™ PLUS Catheters

Features	Benefits
Up to 0.070 Inch Lumen	Large lumen for strong aspiration & capture of large thrombus
Exceptionally Soft Distal Tip	Allows smooth distal navigation and easy passage of bifurcations
Steam Shapeable Tip and Torqueable Shaft	Highly navigable for patients with challenging anatomy
Enhanced Kink Resistance	Maintains lumen integrity during navigation and aspiration in tortuous anatomy

5 Study Procedures

5.1. Screening

Patients presenting with AIS will be evaluated by the physician to establish an appropriate treatment plan based on the patient’s medical condition and available diagnostic screening procedures prior to recruitment in the study. If treatment of the ischemic stroke with a mechanical thrombectomy device is deemed appropriate, the following evaluations will be performed to determine if they meet the inclusion and exclusion criteria. All assessments in the study are made on the basis of good standard of care and not specifically for the study. This applies both to clinical assessments and imaging studies. There are no follow-up requirements for participants who are general eligibility screen failures.

5.2. Informed Consent

Image data acquisition, treatment via interventional procedure with a CE-marked device and clinical evaluation are all considered standard of care and thus there is no need for additional consent. Informed consent for data collection must be obtained prior to enrollment of patients in this study and data transmission.

However, most patients will be unable to give their consent prior to treatment. We expect that approximately 60% of patients will be unable to give informed consent prior to therapy with the SOFIA™/ SOFIA™ PLUS catheter. Excluding this group of patients from the study would entail severe scientific and clinical drawbacks. Results would be biased by systematically excluding more severe strokes and patients with aphasia, i.e. mostly left hemispheric strokes. As a consequence, the generalizability of the study results would be limited.

Once the patient's potential eligibility has been determined, the Investigator will discuss the study and ask the patient, or the patient's legally authorized representative, based on the site routine practice and regulatory requirements, if they are interested in participating in the data collection. The study will be explained to the consenting person in lay terms. Study personnel should document the consent process in the participant's medical record per Good Clinical Practice (GCP). The participant or legally authorized representative is to be provided a copy of the signed ICF. In case the patient was not able to provide his consent before the procedure, all attempts will be made to obtain his consent before discharge. Additionally, patients who signed an Informed Consent Form but did not meet general inclusion/exclusion criteria should also be recorded in the screen failure log.

5.3. Pre-treatment Assessment

Patient history will include but not be limited to the following risk factors and comorbidities:
age, height, weight, gender, hypertension, hyperlipidemia, diabetes mellitus, ischemic heart disease (history of myocardial infarction, angina pectoris, previous stroke, or any cerebrovascular disease), atrial fibrillation, congestive heart failure, peripheral artery disease, current smokers.

- Obtain the time of symptom onset and record it with the time of hospital admission.
- Obtain appropriate non-contrast CT (NCCT and CTA) scans or MRI (and MRA) performed first and record this as well as the time of NCCT/MRI. The ASPECT (or MR DWI-ASPECT) score will be then calculated.
- Neurological examination will include an assessment of the NIH Stroke Scale (NIHSS) within the 2 hours before treatment. NIHSS certified study personnel must determine NIHSS.
- Obtain patient's functional status by assessing his/her modified Rankin Score (mRS prior to stroke onset).

- Physical examination will include recording of supine blood pressure and heart rate.
- Obtain, as routine practice, platelets, glucose and international normalized ratio (INR), partial thromboplastin time (PTT).
- Females who are pregnant or lactating are excluded from the study. A pregnancy test should be ideally administered to all female patients of childbearing potential.
- All potentially stroke impacting concomitant medication will be captured (ASA, anticoagulants, antiplatelet, and hypertensive medications).

5.4. Enrollment

Once all inclusion/exclusion criteria are met, the patient is enrolled when a SOFIA™/ SOFIA™ PLUS Catheter is inserted in the patient and will be assigned a five digit identification number (participant study number). The first two digits assigned represent the site identification, followed by a hyphen and three subsequent digits which represent the sequential enrollment number. For example, ID: 04-002 would represent the second participant enrolled at site 04.

5.5. Procedure

The SOFIA™/ SOFIA™ PLUS Catheter will be used in removal/aspiration of emboli and thrombi following the CE marked Instructions For Use. Enrollment into the study does not change the routine care at the site provided to the patient requiring mechanical thrombectomy treatment.
The time of the following events will be recorded for each patient:

- Index stroke symptom onset
- Hospital admission
- Arterial puncture for endovascular intervention
- Pre-procedure TICI score
- Start of aspiration by the SOFIA™/ SOFIA™ PLUS
- End of aspiration by the SOFIA™/ SOFIA™ PLUS
- Time of first TICI 2b or 3
- If an adjunctive treatment is used, time and TICI score after SOFIA™/ SOFIA™ PLUS Reperfusion Catheter has been used
- Last angiogram taken after all treatments at the end of the procedure.

Additionally, the following information will be recorded:

- Location and length of occlusion
- The final TICI after all adjunctive treatments
- Ancillary device and/or adjunctive treatment trade mark and model
- Intraprocedural complications
- Presence of vasospasm (time of onset, vessels involved, time resolved)
- Evidence of clot migration or embolization

- Dissections/ perforations
- Devices deficiencies, malfunction, complaints (including 2nd line devices in case of aspiration failure)
- Antiplatelet therapy regimen
- Sedation

Those patients with improving neurological status prior to endovascular intervention may or may not undergo mechanical thrombectomy at the discretion of the treating physician.

The definition of rapid improvement that leads to foregoing endovascular intervention is at the discretion of the Investigator based on clinical judgment regarding risks versus benefits.

Those patients excluded according to the selection criteria but treated with a SOFIA™ / SOFIA™ PLUS Catheter will be considered as intent to treat patients.

5.6. Additional Devices or Procedures

There may be circumstances requiring the use of other techniques (mechanical thrombectomy devices or medical therapy) should the SOFIA™ / SOFIA™ PLUS Catheter fail to achieve recanalization in the targeted vessel. **After three (3) unsuccessful aspiration attempts the investigator must** use other techniques. However, the investigator **may decide at any point** to change his/her treatment for optimum patient care. Any devices which are CE-Mark approved for mechanical thrombectomy might be used. Use of these devices after the first aspiration using the SOFIA™ / SOFIA™ PLUS Catheter will not be considered a protocol deviation but will be recorded.

5.7. Angiographic Imaging

At pre-procedure Digital Subtraction Angiography (DSA) shall be performed to define the angioarchitecture of the occluded vascular segment. When possible, an assessment of collateral blood flow by DSA should be done per institutional standard of care, particularly in cases of terminal internal carotid artery occlusion. Prior to mechanical thrombectomy by the Sofia Device, baseline Thrombolysis in Cerebral Infarction (TICI) scores shall be obtained by DSA. CTA or MRA is not an acceptable substitute for this assessment. The Investigator shall make an initial assessment of TICI flow in the target vessel territory. TICI scores are to be assessed after completion of the procedure during the final angiographic run. Pre-procedure and post-procedure (pseudonymized) angiograms shall be sent to an independent Core Laboratory to make a final determination on TICI flow.

5.8. Follow-up at 24 ± 12 Hours Post-procedure

Per institutional standard of care at the site, follow-up imaging (MRI preferred, CT if medically required) shall be performed for all patients at 24 (-12/+12) hours post-stroke to assess

for intracranial hemorrhage and for infarct volume assessment by the Core Lab based on ECASS definitions [25]:

- HI 1 (small petechiae along the margins of the infarcted area without space-occupying effect)
- HI 2 (more confluent petechiae within the infarcted area but without space-occupying effect)
- PH 1 (hematoma in ≤ 30% of the infarcted area with some slight space-occupying effect)
- PH 2 (hematoma in > 30% of infarcted area with substantial space-occupying effect).

A physical exam, as well as clinical and neurological assessments, will be completed per institutional standard of care at 24 (-12/+12) hours post treatment. Data to be collected and then captured on eCRFs include:

- Any adverse events or serious adverse events
- Vital signs (blood pressure and heart rate)
- Significant findings from clinical assessment and physical exam (i.e. all new, worsening, or improved conditions)
- All significant neurological findings, NIHSS and mRS Score (24 (-12/+12) hours), collected during independent neurological assessment by qualified personnel

5.9. Follow-up at Discharge (7±3Days Post-procedure)

Per routine practice at the site, patients will be evaluated for neurological (NIHSS) and disability (mRS) status

- All significant neurological findings, NIHSS and mRS Score
- Any adverse events and serious adverse events will be reported by the site

5.10. Follow-up at 90 ± 14 Days Post-procedure

- Per routine practice at the site, patients will be evaluated for their functional outcome with mRS
- A Quality of Life questionnaire will be conducted with the patient or relative
- Any adverse events and serious adverse events will be reported by the site
- Antiplatelet therapy regimen is recorded throughout the study
- The "Study Completion Form" CRF will be filled in at the end of the study (even if the patient is lost to follow-up before the 90-day assessment, withdrew his or her consent, or is deceased)

5.11. Unscheduled Visits

As per site routine practice, patient safety and study assessments (imaging, NIHSS, mRS) at other follow-up visits should be done as clinically indicated with corresponding data documented in the source documents and appropriate CRFs.

5.12. Termination of Patient Participation

All patients have the right to terminate their participation at any point during the study. In addition, Principal Investigators also have the ability to terminate patient participation in the study. Reasons for termination include: completion of study, patient withdrawal, physician-directed patient withdrawal, lost-to-follow-up, and death. A description of the reason for their termination will be documented in the patient's medical file and in the appropriate study Case Report Forms (CRF).

Withdrawal:

A participant or his/ her legal authorized representative can stop the participant's participation in this study by withdrawing consent, or decide to discontinue participation if the participant is no longer able to participate in the study (owing, concomitant disease or administrative reasons). If a participant or his/ her legal authorized representative decides to withdraw the participant from the study, it can be done at any time. Whenever possible, the reason for discontinuation should be recorded in the source documentation and on the eCRF. Withdrawing consent will not result in any penalty whatsoever for the participant. Further treatment will be performed according to local practice. The data of the participant who withdraws his/her informed consent, collected prior to withdrawal, will not be discarded, and will be used in the final analysis of the study.

Lost to follow-up:

After three documented attempts to reach the participant by telephone have failed, a certified letter will be sent to the participant. The participant will be considered lost to follow-up if this communication is unsuccessful.

Participants who discontinue study participation prematurely will not be replaced.

6 Assessment of Safety and Effectiveness

6.1. Safety

Safety will be assessed by collecting adverse event (AEs) data during the endovascular procedure, at 24 hours post-procedure, at hospital discharge or 7-10 days, and at 90 days after the index procedure.

Adverse events will be ascertained by the investigators using non-leading questions, noted as spontaneously reported by the patients to the medical staff or observed during any measure-

ments on all study days. The observation period begins with start of the endovascular pro-cedure and ends at follow- up at day 90. AEs are also documented at the unscheduled visits, if applicable.

6.1.1. Device Deficiency

A device deficiency is defined as inadequacy of a medical device with respect to its identity, quality, durability, reliability, safety, or performance. Device deficiencies include malfunctions, use errors, and inadequate labeling. All device deficiencies must be reported to the Study Coordinator, the device manufacturer, and, if necessary, the respective site's government agencies.
If a device deficiency results in an adverse event for the patient, this adverse event (AE) will be considered reportable and must be reported as an adverse event in the eCRF; in case of seriousness also on the SAE-form (see 6.1.2) . Device deficiencies that do not result in an adverse event for the patient do not need to be recorded as an AE, as they are not considered an AE. They will be considered as technical event and documented separately.

6.1.2. Adverse Events and Serious Adverse Events

Definition Adverse Event (AE):

Any untoward medical occurrence, unintended disease or injury, or any untoward clinical signs (including an abnormal laboratory finding) in subjects, users or other persons whether or not related to the medical device.

Definition Serious Adverse Event (SAE):

Any event meeting the following criteria will be classified as SAE according to the GCP and ISO 14155 definition:

a) Led to a death,

b) Led to a serious deterioration in health that resulted in:

1) a life-threatening illness or injury, or

2) a permanent impairment of a body structure or a body function, or

3) patient hospitalization or prolongation of existing hospitalization, or medical or surgical in-tervention to prevent life threatening illness or injury or permanent impairment to a body structure or a body function.

4) medical or surgical intervention to prevent life-threatening illness or injury or permanent impairment to a body structure or a body function,

c) Led to fetal distress, fetal death or a congenital abnormality or birth defect.

> *NOTE 1: This includes device deficiencies that might have led to a serious AE (SAE) if suitable action had not been taken, intervention had not been made, or circumstances had been less fortunate. These are handled under the SAE reporting system.*

NOTE 2: A planned hospitalization for pre-existing condition, or a procedure required by the Clinical Investigation Plan, without a serious deterioration in health, is not considered to be an SAE.

This registry study is designed to minimize potential risks to the participants. However, the following events associated with endovascular treatment / thrombectomy should be documented on the eCRF.

Table 3: Events

Event	Collected data
Intracranial hemorrhage (symptomatic /asymptomatic)	Date/time; Symptomatic/asymptomatic
New disabling ischemic stroke (NIHSS increase of at least 4 points)	Date/time; Inside/outside the region of the treated vessel /assignment not possible
New non-disabling ischemic stroke (If NIHSS increase it should be less than 4 points)	Date/time; Inside/outside the region of the treated vessel /assignment not possible
Severe extracerebral hemorrhage (i.e. requiring surgical treatment or transfusion)	Date/time;Gastrointestinal/local (groin) / other
Myocardial infarction	Date/time
Pseudoaneurysm femoral artery	Date/time
TIA	Date/time; Inside/outside the region of the treated vessel /assignment not possible
Death	Date/time

5.1.3. Reporting of SAEs Incidentsby the Investigator to the Sponsor

All SAEs must be reported by the investigator [to the responsible Safety Officer at the KKS Heidelberg] within 24 hours after the SAE becomes known using the "Serious Adverse Event" form.

The reporting will be performed by faxing a completed ‘SAE Form’ to the KKS Heidelberg, fax number:

+49 (0)6221-56-33725

All SAEs incidents will be reported to the national competent authorities, to the ethics committees and other administrative bodies according to the national regulatory requirements by the Safety Officer at the KKS Heidelberg.

6.2. Effectiveness

The effectiveness of the device will be assessed based on angiographic revascularization of the occluded target vessel immediately post-procedure as defined by a TICI 2b or 3 score and at 90 days after the procedure as defined by good functional outcome with a mRS score of 0-2.

6.2.1. Functional Outcome

Good patient functional outcome at 90 day follow-up will be defined by a mRS score of 0-2.

6.2.2. Angiographic Outcome

Angiographic revascularization of the occluded target vessel immediately post-procedure as defined by a TICI 2b or 3 score assessed by an imaging corelab

*Table 4 - Thrombolysis in Cerebral Infarction (TICI) Perfusion Categories***

Grade 0	**No perfusion** or anterograde flow beyond site of occlusion
Grade 1	**Penetration but not perfusion.** Contrast penetration exists past the initial obstruction but with minimal filling of the normal territory
Grade 2	**Incomplete perfusion** wherein the contrast passes the occlusion and opacifies the distal arterial bed but rate of entry or clearance from the bed is slower or incomplete when compared to non-involved territories
Grade 2A	**Some perfusion** with distal branch filling of <50% of territory visualized
Grade 2B	**Substantial perfusion** with distal branch filling of ≥ 50% of territory visualized
Grade 2C	**Near complete perfusion** except for slow flow/occlusion in 1 or 2 branches of few distal cortical vessels, or presence of small distal cortical emboli
Grade 3	**Complete perfusion** with normal filling of all distal branches

** From Almekhlafi et al. Interv Neuroradiol. 2014 Jan-Feb;20(1):21-7. Epub 2014 Feb 10.

The Investigator shall make an initial assessment of TICI flow in the target vessel territory. Pre-procedure and post-procedure pseudonymized angiograms shall be sent to an independent Core Laboratory to make a final determination on TICI flow.

6.3. Risk/Benefit Analysis

The risks associated with the Sofia/Sofia Plus Catheter as an adjunct to IV rtPA are similar to those associated with cerebral angiography and with other intra-arterial methods of recanalization.

6.3.1. Angiography

An angiographic procedure requires that a catheter be placed into the body, and in this case, threaded through the body to the neurovasculature. Once the catheter is in the proper position, contrast media is infused through the catheter to examine the vessels. Patients may encounter bleeding from vessel perforation, vessel spasm, swelling, or bruising at the access site where the catheters are placed in the body (usually the groin area). Additionally, once the catheter reaches the neurovasculature, there is a chance that the catheter could cause bleeding, hematoma, vessel thrombosis, dissection, distal embolization, pseudoaneurysm, and arteriovenous fistula formation. Patients who are allergic to contrast media are at further risk and may experience a reaction that may include hives, itching, nausea, or breathing difficulty. Contrast media may also cause kidney damage in some patients. The above risks of angiography are well known and generally unlikely to occur. However, if any of the above risks occur and are severe enough, patient death is possible.

6.3.2. Computed Tomography Scan (CT Scan) and Magnetic Resonance Imaging (MRI)

The risks encountered during a CT scan are low. The risks mentioned above for contrast media allergy are possible with a CT scan. There is also a slight risk of injury from being exposed to the radiation associated with a CT scan; however, the levels of radiation, and the risks associated with radiation exposure are low. MRI does not use ionizing radiation. There are no known harmful side-effects associated with temporary exposure to the strong magnetic field used by MRI scanners. These exams are performed according to usual routine practice at the site, for such stroke patients admitted and treated accordingly.

6.3.3. Thrombectomy/Embolectomy

The risks of intra-arterial thrombectomy/embolectomy are similar to those associated with existing intra-arterial methods of recanalization. These risks include:

- allergic reaction and anaphylaxis from contrast media
- acute occlusion
- hematoma or hemorrhage at access site
- infection
- inability to completely remove thrombus
- air embolism

- arteriovenous fistula
- ischemia
- kidney damage from contrast media
- death
- neurological deficits including stroke
- device malfunction
- distal embolization
- intracranial hemorrhage
- vessel spasm, thrombosis, dissection
- false aneurysm formation

Risks directly associated with the Sofia/Sofia Plus Catheters are similar to the above. Considerable testing has been completed to ensure that the Sofia/Sofia Plus Catheters do not pose a significant risk. Testing includes both in vitro and in vivo testing. In addition, the following will be performed to minimize the risks:

- As per usual site practice, patients will be carefully evaluated before entering the study to ensure that the location of the occlusion and the time of stroke onset are appropriate for treatment with the Sofia/Sofia Plus Catheters.
- During the clinical study, the procedure will be performed in an Operating Room or in an angiographic suite with an Operating Room standby. Therefore, should complications arise requiring surgery or other interventions, the surgery or intervention can be initiated without delay. All sites will be carefully selected to ensure that either a stroke unit operating according to national stroke guidelines or a physician experienced in treating patients presenting with acute ischemic stroke is available.
- Patients will be carefully monitored as per site routine practice and the follow-up period. The Investigator will examine and perform various diagnostic tests before, during, and after the procedure, at 7-10 days or at hospital discharge and at 90 days (±14 days) after the procedure.

Use of the Sofia/Sofia Plus Catheters offer several potential benefits, which may include a higher success rate of recanalization with an acceptable device-related serious adverse rate. Based on the above information, the benefits of the Sofia/Sofia Plus Catheters outweigh the potential risks associated with their clinical use.

7 Imaging Core Lab

For all study participants, the imaging core laboratory will perform the qualitative and semi-quantitative assessments of vessel occlusion (site of occlusion, length of occlusion, TICI score) and ASPECT score at baseline and follow-up as well as revascularization at the end of the procedure (TICI score). Furthermore, ICH and infarct volume assessment at 24 (-/+ 12) hours using clin-

ical routine data will be performed. All sites must provide the complete routine imaging (as per local standard of care), and all images must be de-identified prior to submission (pseudonymisation). A detailed imaging core lab manual will be provided.

8 Data Management

Clinical site personnel will enter data into eCRFs in the EDC system provided by the Study Coordinator for study data collection. The Study Coordinator or designee will be responsible for confirming the overall integrity of the data. The image data workflow will be described more extensively in the imaging core lab manual.

8.1. Data Prottection

The data obtained in the course of the trial will be treated pursuant to the EU Data Protection Regulation (EU Datenschutz-Grundverordnung (DSGVO)).

During the clinical trial, patients will be identified solely by means of their individual identification code (patient number). Trial findings stored on a computer will be stored in accordance with local data protection law and will be handled in strictest confidence. For protection of these data, organizational procedures are implemented to prevent distribution of data to unauthorized persons. The appropriate regulations of local data legislation will be fulfilled in its entirety.

The patient consents in writing to release the investigator from his/her professional discretion in so far as to allow inspection of original data for monitoring purposes by health authorities and authorized persons (inspectors, monitors, auditors). Authorized persons (clinical monitors, auditors, inspectors) may inspect the patient-related data collected during the trial ensuring the data protection law.

The investigator will maintain a patient identification list (patient numbers with the corresponding patient names) to enable records to be identified.

Patients who did not consent to circulate their pseudonymized data will not be included into the trial.

This protocol, the eCRFs and other trial-related documents and material must be handled with strict confidentiality and not be disclosed to third parties except with the express prior consent of Sponsor. Staffs of the investigators involved in this study are also bound by this agreement.

9 Statistical Methods

9.1. Sample Size Justification

Sample size: 250 patients

Within a descriptive study, the number of participants required depends on the desired accuracy for observed frequencies. With 236 patients enrolled in the cohort, a 95% confidence interval

size of ± 6.0% will be obtained for a attended frequency of mRS≤2 at 90 days of 32,6% (rate observed in Mr Clean). In addition, even with 5% of patients lost to follow-up, we still dispose of an accuracy of at least ±6% for observed frequencies (Effectiveness and safety parameters).

9.2. Statistical Analysis

The objective of the statistical analyses is to support the demonstration that the Sofia/Sofia Plus Catheters used as aspiration catheter can reach Clinical outcome defined as mRS ≤2 at 90 days. All confidence intervals presented will be two-sided. All statistical tests will be two-tailed with a significance level of 0.05. Descriptive statistics will be provided. This includes the number of observations, mean, median, standard deviation, minimum and maximum for continuous variables and counts and percent for discrete variables. Results collected at multiple visits will be summarized at each visit. Summaries for all measures will include all observed data for each visit. All endpoints will be evaluated for the SOFIA and SOFIA Plus Catheter separately.

9.2.1. Definition of Analysis Samples

All primary and secondary Effectiveness and safety outcome measures will be analyzed under both the intent-to-treat (ITT) and per-protocol (PP) principles.

a) Intent to Treat Sample

The ITT sample includes all participants who are enrolled.

b) Per Protocol Sample

The per-protocol sample will include all participants that do not have the following protocol violations or deviations:

1) Eligibility violation

2) No Sofia/Sofia Plus treatment performed

3) mRS primary effectiveness only: missing 90-day outcome (not including missing due to death prior to the 90 days)

9.2.2. Effectiveness

Primary Endpoints

The primary effectiveness variable is the Clinical outcome defined as mRS ≤ 2 at 90 days (assessment performed by an independent neurologist)

The proportion of patients who are successful based on each criterion will be calculated. Estimates of the group differences and their 95% confidence intervals will be calculated. Multiple ordinal logistic regression analysis estimated the effect of treatment and tested for the interaction of time to reperfusion with treatment. The effect of treatment as a risk difference on reaching independence (mRS score, 0-2) will be computed as a function of time to reperfusion. Whenever appropriate, calculations will be adjusted for age, National Institutes of Health Stroke Scale score, previous

stroke, atrial fibrillation, diabetes mellitus, smoking status and intracranial arterial terminus occlusion. For missing data (patients who were lost to follow-up) the mRS scores at 90 days will be assigned using multiple imputations, worst case scenario (mRS = 6), and best case scenario (mRS=0).

9.2.3. Safety

The safety variables:

- The occurrence of major neurological events (stroke, intracranial hemorrhage, intracerebral hemorrhage, etc.) prior to discharge
- Devices and procedure related adverse events within 90 days of follow up
- Occurrence of embolization in previously uninvolved (or new) territories (ENT) as seen on the final control angiogram at the end of the procedure
- Occurrence of symptomatic intracranial hemorrhages (sICH) within 24 hours
- Occurrence of intracranial vessel damages at the end of the procedure.

Descriptive statistics will be provided for each safety variable. Estimates of the group differences and their 95% confidence intervals will be calculated.

9.2.4. Recanalization:

- Proportion of patients having complete recanalization (TICI ≥ 2b) just after first line aspiration treatment compared to historic controls based on published RCTs*
- Proportion of patients having complete recanalization (TICI ≥ 2b) after thrombectomy using an additional device
- Time from groin puncture to complete recanalization (TICI ≥ 2b) in patients after first line aspiration treatment (SESAME).
- Time from groin puncture to recanalization (TICI ≥ 2b) after thrombectomy using an additional device
- Time from CT-scan/MRI at the institution to groin access
- Time from symptom onset to CT-scan/MRI

* assessed by an imaging core lab.

Descriptive statistics will be provided for each recanalization measurement variable. Estimates of the group differences and their 95% confidence intervals will be calculated.

9.2.5. Clinical endpoints / quality of life:

- NIHSS score at 24 hours
- NIHSS score at discharge
- Quality of Life at 90 days

Descriptive statistics will be provided for quality of life variables and changes in variable values. Estimates of the group differences and their 95% confidence intervals will be calculated.

9.2.6. Supplemental Endpoints

Imaging endpoints:

- Difference of ASPECT scores in CT/MRI pretreatment vs. 24h
- In the subgroup of patients with additional perfusion CT (as per local standard of care): volume of saved brain tissue determined by predictive modeling

Health Economics:

- Device costs (standardized cost of all devices as well as human resources and medication used during index procedure)
- Hospital length of stay

Descriptive statistics will be provided for supplemental endpoints. Exploratory data analysis will be performed to assess the possible influence of treatment factors on health economic measures. Estimates of the group differences and their 95% confidence intervals will be calculated.

9.2.7. Analysis of Adverse Events:

All adverse events will be summarized by showing the number and percent of patients reporting the event. Events will also be reported by relationship to the procedure or device.

9.2.8. Interim Analysis and Early Stopping Rules

An interim analysis is planned after the first 50 patients enrolled have reached the 3 months follow-up for the purpose of assessing Effectiveness and safety tendencies. If interim analysis data are sufficient to demonstrate study success, then the trial enrollment will be terminated early. If interim analysis data indicate that study success is highly unlikely even if enrollment were to continue to a maximum sample size of 250, then the trial enrollment will be terminated early for futility. Otherwise, study enrollment will continue to a maximum sample size of n=250 and analysis will be performed when 90-day follow up visits are complete.

The decision to stop or continue the trial will be based on the overall assessment of risk and benefit by the steering committee, supported by the CEC. Safety interim analysis will be performed after one third and two thirds of the patients have been included.

10 Quality Control and Quality Assurance

10.1. Training of Investigator and Site Personnel

The training of the Investigator, and appropriate clinical site personnel will be the responsibility of the Study Coordinator and PI, or designee, and may be conducted during an Investigator meeting, a site initiation visit, or other appropriate training sessions. To ensure proper device usage,

uniform data collection, and protocol compliance, the Study Coordinator or designee will present a formal training session to study site personnel which will review the Instructions For Use of the device, the Investigational Plan, instructions on data collection, schedules for follow-up with the study site coordinators, and regulatory requirements. Detailed feedback regarding completion of forms will be provided by the Study Coordinator or designee through the regular site monitoring.

In order to provide for safe use of the devices the primary concern in operator selection for this study is adequate experience, commitment to safety, and consistency in adherence to the clinical protocol. Therefore, the interventional investigators selected to participate will be neurointerventionalists who, by virtue of their experience and training, are accomplished in cerebral flow restoration techniques using neurothrombectomy devices in intracranial arteries. Each interventional investigator will be experienced, and will have participated in at least 20 for direct aspiration as a first line treatment technique procedures; documentation of experience to be provided before or during site initiation visit.

10.2. Data Monitoring

The Study Coordinator is responsible for ensuring that the study is conducted according to the appropriate regulations. An employee of the Study Coordinator or designate will conduct the following site visits:

10.2.1. Site Qualification

A questionnaire will be sent to each site wishing to participate in order to ensure the investigational site has the appropriate staff, facilities, and expertise to participate in the study.

10.2.2. Site Initiation Visit

Conducted to train the investigational staff on use of the device, study requirements, and other relevant training. The Study Coordinator or a representative will conduct site initiation visits at each investigational site before enrollment begins at that site.

10.2.3. Data Monitoring Site Visit

Conducted as needed to ensure the investigational site is operating in compliance with EN ISO 14155:2011, GCP guidelines, and this protocol, continues to have the appropriate staff and facilities, and is correctly completing the Case Report Forms (CRFs) (verification against source data).

To ensure that Investigators and their staff understand and accept their defined responsibilities, the Study Coordinator will maintain regular correspondence and perform periodic site visits during the course of the study to verify the continued acceptability of the facilities, compliance with the investigational plan, and maintenance of complete records. Clinical monitoring will include review and resolution of missing or inconsistent data and source document checks to ensure the accuracy of the reported data. CRFs for all enrolled patients will be made available to the Study

Coordinator for review and collection as agreed with the Investigator. The Study Coordinator will evaluate and summarize the results of each site visit in written reports, identifying repeated data problems with any Investigator and specifying recommendations for resolution of noted deficiencies. The first monitoring visit will be conducted at each site after inclusion of the first patients at each site in order to ensure that all aspects of the protocol are followed as well as to accurately record results, report adverse events, and keep records.

The next periodic monitoring will be performed remotely by the Study Coordinator or its representatives, depending on the enrollment rate at the site and occurrence of adverse events, deviations or any other relevant topics. At least one on-site monitoring visit will be performed before the close-out visit, even if the site does not enroll as many patients as planned.

10.2.4. Study Close-Out Site Visit

Conducted at the termination of this study to resolve any outstanding data queries, and to ensure that any remaining study materials are returned to the Study Coordinator.

10.3. Investigator Requirements

All Investigators must submit the following documentation to be considered approved Investigators:

1) Signed and dated recent curriculum vitae
2) Signed Clinical Study Agreement (CSA)
3) Complete site qualification process and site initiation

The Study Coordinator will make the final determination of activated Investigators. Investigators must allow the Study Coordinator or its representatives to visit the site to periodically assess the data quality and study integrity. On site, the Study Coordinator or its representatives will review study records in comparison with source documents, discuss the conduct of the study and verify that the facilities remain acceptable. In addition, the study may be evaluated by government inspectors who must be allowed access to CRFs, source documents, and other study files.

The Investigator must notify the Study Coordinator promptly of any inspections scheduled by regulatory authorities, and promptly forward copies of inspection reports to the Study Coordinator.

10.3.1. Ethics and Regulatory Considerations

This study will be carried out according to the study protocol, the Declaration of Helsinki from October 2013, the German Medical Device Act (MPG), whereas the § 23b will apply, and the guidelines of ICH-Verordung as well as the General Data Protection Regulation (EU 2016/679).

Participation in this study is voluntary. The decision to participate or to decline participation will not affect the patient's treatment in any way.

A participant or his/her legal authorized representative can stop the participant's participation in this study by withdrawing consent, or decide to discontinue participation if participant is no longer able to participate in the study (owing to adverse events, concomitant disease or administrative reasons). If a participant or his/her legal authorized representative decides to withdraw the participant from the study, it can be done at any time. However, the reason for discontinuation must be recorded in the source documentation and on the eCRF.

The data of the participant who withdraws his/her consent will be discarded unless the patient permits further use. In the latter case , data will be used in the final analysis of the study. During the consent procedure the participant or his/her legal representative have the possibility to agree or decline any further use of the data should consent be withdrawn. Furthermore, should the participant or his/her legal representative change their mind, data removal can be requested at any time, and his /her data will be destroyed and not be integrated in the statistical analysis. However, data removal will no longer be feasible once the data has been completely anonymized.

Also, the investigator can decide on the study termination of the participant in case of AEs which make the continuation not desirable, major protocol deviations, or non-compliance of the participant.

Withdrawing consent will not result in any penalty whatsoever for the participant. Further treatment will be performed according to local practice. In case of a device-related or procedural-related adverse event that occurred as a result of participating in this study, the participant will be treated according to local practice.

Prior to enrolling patients into the study, the Investigator will ensure that proper Ethics Committee (EC) approval is obtained. The EC shall approve all study documents as appropriate, including the final protocol, amendments to the protocol, Instruction for use and the Informed Consent Form.

The investigator will promptly report to the EC/RB Board all changes in activity and all unanticipated problems involving risks to human participants or others, and will not make any changes in the research without completing any necessary EC/IRB approval steps, except when necessary to eliminate immediate hazards to human participants.

The investigator must report to the EC/IRB at least yearly on the progress of the investigation, if required. A letter from the EC/IRB should document continuing EC/IRB review. Notification to the EC/IRB by the investigator within 3 months after completion, termination, or discontinuation of the study at the specific site must be documented.

Other investigator responsibilities to the EC/IRB and Study Coordinator include the following:

- During the conduct of the study, submit progress reports to the EC/IRB as required.

- As required, obtain approval from the EC/IRB for protocol amendments and for revisions to the informed consent or participant recruitment advertisements. A copy of the correspondence should be provided to the study monitor.
- Notify the EC/IRB (if required) and Study Coordinator of any protocol deviation to protect the life or physical well-being of a participant in an emergency within 24 hours but in no instance later than 5 days after the emergency occurred.
- Provide EC/IRB with any other information it requests before or during the conduct of the study.
- Maintain a file of study-related information that includes all correspondence with the EC/IRB
- Notify EC/IRB within 3 months after study completion, termination or discontinuation.
- Notify Study Coordinator, within 24 hours, of withdrawal of approval by the reviewing EC/IRB.

Informed Consent

Informed consent has to be obtained prior to enrolment of patients in SESAME. In the normal case written consent will be given by the patient after detailed information about the clinical trial by the investigator. The investigator or sub-investigator will explain the study in detail including risks and benefits for the patient, financing of the study and potential conflicts of interest. Furthermore, the investigator will clarify that the patient or his/her legal guardian will document his/her agreement to participate in the SESAME study by signing the informed consent form.

However, given the sample to be studied (i.e. acute stroke patients showing neurological deficits that may comprise aphasia or disturbance of consciousness) we expect that a certain proportion of patients (estimated 60%) will be unable to give informed consent. Excluding this group of patients from the clinical trial would entail severe scientific and clinical drawbacks. Results would be biased by systematically excluding more severe strokes and patients with aphasia, i.e. mostly left hemispheric strokes. As a consequence, the generalizability of the study results would be limited. Strictly speaking, in case of a positive result this could not be transferred to the patients excluded from the trial (e.g. aphasic patients). This large group would still be excluded from this effective treatment.

A possible solution would allow the ***enrolment of patients unable to give informed consent*** based on their alleged will following pre-specified criteria. These criteria would have to consider a potential individual benefit as well as any possible risk of participation in the clinical trial. In general the protection of the patient and his autonomy has to be weighed against the potential individual benefit as well as the scientific interest. Following European and national regulations the enrolment of patients (temporarily) unable to give informed consent into clinical trials is possible in an emergency situation if a legal guardian is available and – after careful consideration of possible risks and potential individual benefit – a participation in the clinical trial would comply with

the patient's presumptive will. Moreover, standard treatment following best medical practice must not be withheld to the patient by enrolment in the clinical trial.

For patients eligible for enrolment in SESAME best medical practice in these patients usually comprises the use of CT to rule out hemorrhage or prove ischemic infarction followed by an interventional procedure (thrombectomy), hospitalization and monitoring in a specialized stroke unit. Additional standard treatment consists of supervision and modification of physiological parameters (such as blood pressure, body temperature, blood glucose) and early secondary prevention (e.g. Aspirin).

Suggestion for enrolment of patients unable to give informed consent in SESAME:

A – Patient is able to give informed consent and able to provide written consent:

Patient is informed by investigator, patient information is handed over to the patient, and patient provides written consent. This may occur prior to treatment or after treatment if the patient was unable to give informed consent and underwent standard of care treatment in agreement with the protocol.

B – Patient is unable to give informed consent, legal guardian is available

Legal guardian acts on behalf of the patient; legal guardian is informed by investigator, patient information is handed over to the legal guardian, legal guardian provides written consent

C –Patient is unable to give informed consent; legal guardian is not available, enrolment of patient by consensus between investigator and independent physician

If the patient is unable to give informed consent and no legal guardian has been appointed, the patient may be enrolled by consensus between the investigator and an independent physician about an the presumed will of the patient; if possible, patient's next of kin should be contacted to appraise the patient's presumed will; the decision has to be documented using a special form. Immediately parallel an application for appointing a legal guardian will be started later patient or legal guardian will be informed by the investigator and may or may not provide written consent. Enrolment through the independent physician will be included to minimize selection bias.

Figure 1: "Overview of Ways of Obtaining Informed Consent"

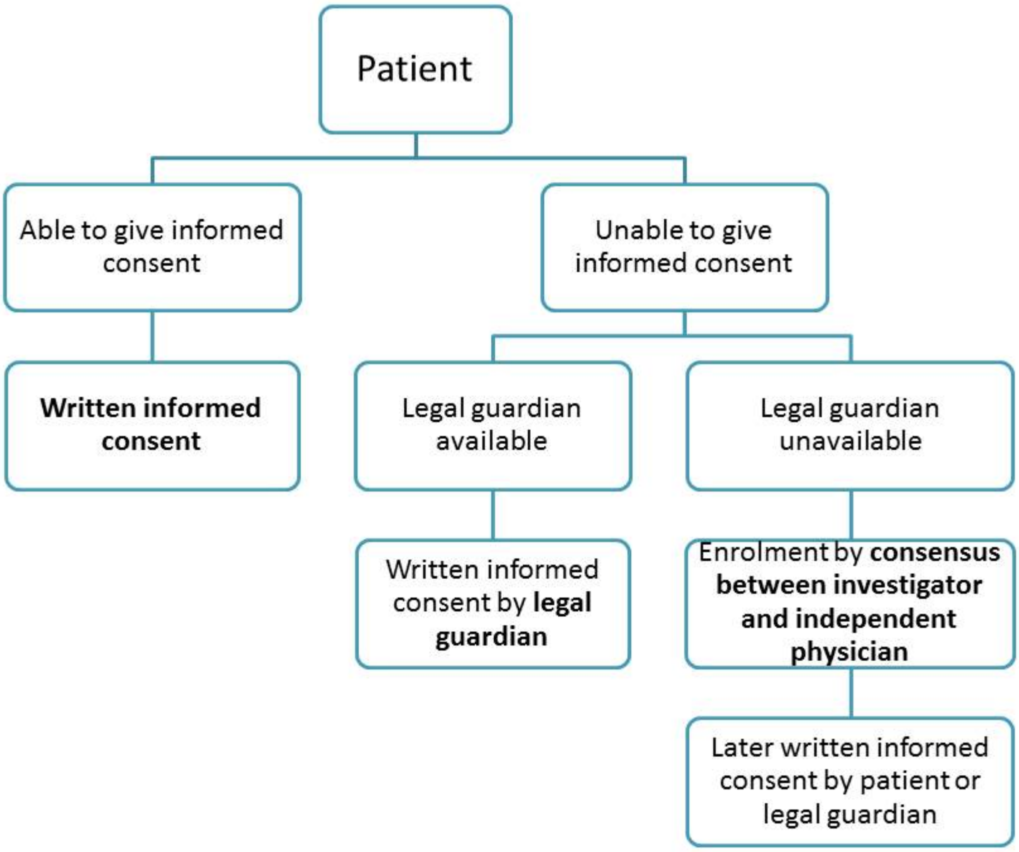

These options for obtaining the informed consent are NOT equal or interchangeable. The highest priority will always be to obtain informed consent by the participant himself. Only if this option is not possible, other ways may be approached.

Data collection will not start prior to informed consent but may be done retrospectively. All information and data are subject to medical professional confidentiality, including patient's names, other confidential data as well as sensitive medical information. Data sent to Study Coordinator or Core Labe concerning participants or their participation in this study will be considered confidential. All information and data captured in EDC system or sent to Study Coordinator will be de-identified information. All data used in the analysis and reporting of this evaluation will be used in a manner without identifiable reference to the participant. The Principal Investigator consents to visits by the staff of the Sponsor and its authorized representatives as well as regulatory authorities, which governs the conduct of clinical investigations.

Data will be fully anonymized as soon as legally and practically (for study purposes) possible.

Study Financing and Conflicts of Interest

Financial support for the SESAME study will be pro MicroVention Europe, a French limited liability company, with capital of 40 000€, registered with the Registry of Commerce and Companies in

Versailles under number 440 775 674 RCS, with registered address at 30 bis, rue du Vieil Abreuvoir, 78100 Saint-Germain en Laye, France.
The financing of the study will provide case fees to reimburse investigators and study nurses for data collection at the study sites as well as central administrative costs such as project management, study monitoring, data managment, CEC meetings etc.. Study participants will not be reimbursed or receive any other incentives for study participation. Similarly, investigators will not receive any incentives for enrolling participants into the study.
MicroVention Europe will have no access to the study participant data and will not be involved in patient recruitment, study design or analysis and interpretation of the data. However, the Study Coordinator will provide regular updates about the study progress including fully anonymized patient statistics to MicroVention Europe.
Publication of study results will disclose financial support.

10.3.2. Protocol Amendment and Modifications

All items in this protocol are to be followed exactly. If an amendment is required, this must be made in written form and receive approval from all persons and authorities who approved the original protocol. Administrative changes (not affecting participants' benefits/ risks ratio) may be inserted with abbreviated approval by the EC/ IRB. Major amendments require full approval of the EC/ IRB and Study Coordinator prior to implementation, except in emergency situations. All amendments will be distributed to all protocol recipients with instructions. Documentation of all correspondence regarding protocol amendment study activity should be forwarded to the Study Coordinator.
In situations requiring departure from the protocol, the investigator or other physician in attendance will contact the Study Coordinator. If possible, this contact will be made before implementing any departure from the protocol. In all cases, contact with the Study Coordinator must be made as soon as possible in order to discuss the situation and agree on an appropriate course of action. The protocol deviation eCRF and source document will describe any departure from the protocol and the circumstances.
It is the responsibility of investigators to inform their EC/ IRB of all protocol amendments or modifications and protocol deviations as required by their EC/ IRB procedure and country law.

10.4. Clinical Events Committee

The Clinical Events Committee (CEC) is made up of independent medical doctors who are not participants in the study. At the onset of the study, the CEC will establish explicit rules outlining the minimum amount of data required, and the algorithm followed in order to classify a clinical event. All members of the CEC will be blinded to the primary results of the trial. The CEC will meet regularly to review and adjudicate any clinical events, to evaluate their relationship to the de-

vice/procedure and to the study endpoints. The CEC will also review and rule on all deaths that occur throughout the trial. A CEC Charter, generated by the CEC membership will identify the frequency of meetings, identify criteria for early review safety, any additional criteria for stopping the study early and criteria for inactivating sites for excessive protocol deviations.

10.5. Corelab Imaging Review

This study will utilize an independent Core Laboratory ("core lab") to adjudicate all angiographic outcomes. For all study participants, the imaging core laboratory will perform the qualitative and semi-quantitative assessments for vessel occlusion (site of occlusion, length of occlusion, TICI score) and ASPECT score at baseline as well as revascularization at the end of the procedure (TICI score) blinded to the treatment arm. Furthermore, ICH and infarct volume assessment at 24 (-/+ 12) hours using clinical routine data will be performed. All sites must provide imaging that is suitable for analysis, and all images must be de-identified prior to submission (pseudonymisation).

11 Appendix

11.1. NIHSS Form

11.2. mRS Definitions

12 Bibliographie

1. Writing Group, M., et al., Heart Disease and Stroke Statistics-2016 Update: A Report From the American Heart Association. Circulation, 2016. **133**(4): p. e38-60.
2. Campbell, B.C., et al., Safety and Effectiveness of Solitaire Stent Thrombectomy: Individual Patient Data Meta-Analysis of Randomized Trials. Stroke, 2016. **47**(3): p. 798-806.
3. Goyal, M., et al., Endovascular thrombectomy after large-vessel ischaemic stroke: a meta-analysis of individual patient data from five randomised trials. Lancet, 2016. **387**(10029): p. 1723-31.
4. Behme, D., et al., Multicenter clinical experience in over 125 patients with the Penumbra Separator 3D for mechanical thrombectomy in acute ischemic stroke. J Neurointerv Surg, 2016. **8**(1): p. 8-12.
5. Broderick, J.P., et al., Endovascular therapy after intravenous t-PA versus t-PA alone for stroke. N Engl J Med, 2013. **368**(10): p. 893-903.
6. Berkhemer, O.A., et al., A randomized trial of intraarterial treatment for acute ischemic stroke. N Engl J Med, 2015. **372**(1): p. 11-20.
7. Jovin, T.G., et al., Thrombectomy within 8 hours after symptom onset in ischemic stroke. N Engl J Med, 2015. **372**(24): p. 2296-306.
8. Saver, J.L., et al., *Stent-Retriever Thrombectomy for Stroke.* N Engl J Med, 2015. **373**(11): p. 1077.
9. Smith, W.S., et al., Mechanical thrombectomy for acute ischemic stroke: final results of the Multi MERCI trial. Stroke, 2008. **39**(4): p. 1205-12.
10. Goyal, M., et al., Analysis of Workflow and Time to Treatment and the Effects on Outcome in Endovascular Treatment of Acute Ischemic Stroke: Results from the SWIFT PRIME Randomized Controlled Trial. Radiology, 2016. **279**(3): p. 888-97.
11. Liebeskind, D.S., et al., Early arrival at the emergency department is associated with better collaterals, smaller established infarcts and better clinical outcomes with endovascular stroke therapy: SWIFT study. J Neurointerv Surg, 2016. **8**(6): p. 553-8.
12. Turk, A.S., et al., Comparison of endovascular treatment approaches for acute ischemic stroke: cost effectiveness, technical success, and clinical outcomes. J Neurointerv Surg, 2015. **7**(9): p. 666-70.
13. Turk, A.S., 3rd, et al., An investigation of the cost and benefit of mechanical thrombectomy for endovascular treatment of acute ischemic stroke. J Neurointerv Surg, 2014. **6**(1): p. 77-80.
14. Rozeman, A.D., et al., Evolution of Intra-arterial Therapy for Acute Ischemic Stroke in The Netherlands: MR CLEAN Pretrial Experience. J Stroke Cerebrovasc Dis, 2016. **25**(1): p. 115-21.
15. Mazighi, M., et al., Impact of onset-to-reperfusion time on stroke mortality: a collaborative pooled analysis. Circulation, 2013. **127**(19): p. 1980-5.
16. Turk, A.S., et al., ADAPT FAST study: a direct aspiration first pass technique for acute stroke thrombectomy. J Neurointerv Surg, 2014. **6**(4): p. 260-4.
17. Turk, A.S., et al., Initial clinical experience with the ADAPT technique: a direct aspiration first pass technique for stroke thrombectomy. J Neurointerv Surg, 2014. **6**(3): p. 231-7.
18. Delgado Almandoz, J.E., et al., Comparison of clinical outcomes in patients with acute ischemic strokes treated with mechanical thrombectomy using either Solumbra or ADAPT techniques. J Neurointerv Surg, 2015.
19. Kabbasch, C., et al., First-line lesional aspiration in acute stroke thrombectomy using a novel intermediate catheter: Initial experiences with the SOFIA. Interv Neuroradiol, 2016. **22**(3): p. 333-9.
20. Lapergue, B., et al., A Direct Aspiration, First Pass Technique (ADAPT) versus Stent Retrievers for Acute Stroke Therapy: An Observational Comparative Study. AJNR Am J Neuroradiol, 2016.
21. Goyal, M., et al., Randomized assessment of rapid endovascular treatment of ischemic stroke. N Engl J Med, 2015. **372**(11): p. 1019-30.
22. Campbell, B.C., et al., Endovascular therapy for ischemic stroke with perfusion-imaging selection. N Engl J Med, 2015. **372**(11): p. 1009-18.

23. Saver, J.L., et al., Stent-retriever thrombectomy after intravenous t-PA vs. t-PA alone in stroke. N Engl J Med, 2015. **372**(24): p. 2285-95.
24. Fransen, P.S., et al., Time to Reperfusion and Treatment Effect for Acute Ischemic Stroke: A Randomized Clinical Trial. JAMA Neurol, 2016. **73**(2): p. 190-6.
25. Berger, C., et al., Hemorrhagic transformation of ischemic brain tissue: asymptomatic or symptomatic? Stroke, 2001. **32**(6): p. 1330-5.
26. Chow, S.S., J; Wang, H, *Sample Size Calculations in Clinical Research*, in *Biostatistics Series*, C. Hall/CRC, Editor. 2008. p. 89.
